# Molecular characterization of BCoV infecting vaccinated and non-vaccinated cattle in Thrace district Türkiye and isolation of field strains

**DOI:** 10.1186/s12985-025-03010-3

**Published:** 2025-12-01

**Authors:** Semaha Gul Yilmaz, Aysun Yilmaz, Gizem Karadag, Nuri Turan, Juergen Richt, Huseyin Yilmaz

**Affiliations:** 1 Department of Virology, Veterinary Faculty, Istanbul University-Cerrahpasa, Hadimkoy, Istanbul, Türkiye; 2 Department of Diagnostic Medicine and Pathobiology, College of Veterinary Medicine, Kansas State University, Manhattan, USA; 3 Department of Veterinary Tropical Diseases, Faculty of Veterinary Science, University of Pretoria, Onders- 10 Tepoort 0110, Pretoria, South Africa

**Keywords:** Bovine coronavirus, Real-time RT-PCR, Phylogenetic analysis, Vaccination, Türkiye

## Abstract

**Background:**

Bovine coronavirus (BCoV) causes neonatal calf diarrhea (CD), winter dysentery in young cattle (WD), and respiratory system infections in cattle of all ages worldwide. The aim of this study was the detection, isolation, and investigation of the frequency, and molecular characterization of circulating BCoVs in cattle in the Thrace district of Türkiye. It was also aimed to determine genotypes and variants to include in vaccine and vaccination strategies.

**Methods:**

For this purpose, a total of 47, including 12 integrated and 35 small-scale farms located in the Thrace district bordering European Union, were visited. A total of, 281 samples, consisting of 189 nasal/oropharyngeal and 92 fecal swabs were collected from calves exhibiting diarrhea and respiratory signs. RNA was extracted from samples and SYBR-Green real-time RT-PCR was performed. Phylogenetic and heatmap analyses were made using the sequences obtained from RT-PCR. Virus isolation was performed in HRT-18 cells using trypsin.

**Results:**

BCoV RNA was detected in 22.06% (62/281) of swabs collected from calves under 6 months with enteric and/or respiratory signs. Detection rates were 21.69% (41/189) in nasal/oropharyngeal and 22.82% (21/92) in fecal swabs. The virus was identified in 95.7% (45/47) of sampled farms. Statistical analysis showed no significant association between BCoV positivity and the vaccination status of animals. Phylogenetic studies of S1 gene region have indicated that the samples detected in the Thrace district were clustered within the GIb subgroup along with European strains (97.6–100%, nucleotide similarity). These samples also exhibited high nucleotide similarity with HECV-4408 from human CoVs (98.28–99.14%) and DcCoV-HKU23 from other animal CoVs (97.85–99.14%). Despite being collected from distinct regions and years, two field isolates exhibited complete nucleotide identity. BCoV was successfully isolated from both nasal/oropharyngeal and fecal swabs.

**Conclusions:**

This study indicates that BCoV remains a health concern in calves in Türkiye, with isolates clustering in the GIb subgroup alongside European strains. Strengthening biosecurity and regular monitoring should remain priorities; collecting immunologic and field-effectiveness data will help guide the development of regionally updated vaccination strategies. A One Health approach that combines full-genome sequencing with epidemiological studies would help assess zoonotic potential.

**Supplementary Information:**

The online version contains supplementary material available at 10.1186/s12985-025-03010-3.

## Background

Bovine coronavirus (BCoV) can infect many ruminant species, odd-toed non-ruminant species, dogs and humans [1–5] causing significant health problems and economic losses worldwide including Türkiye [6–8]. It was first discovered in 1972 by Mebus and colleagues at the University of Nebraska [9]. BCoV infections in cattle can lead to respiratory and digestive system issues, manifesting as neonatal calf diarrhea (NCD) in newborns, winter dysentery (WD) in adult cattle, respiratory disorders in cattle of all ages (Bovine respiratory disease complex-BRDC), and shipping fever [4, 10]. These infections result in calf mortality, impaired growth in calves and cattle, decreased milk production and quality in dairy cattle, and reduced meat yield in beef cattle due to anorexia [11, 12] causing significant economic losses in the livestock sector [6].

Bovine coronavirus is an enveloped, single-stranded RNA virus with positive polarity, a member of the subgenus *Embecovirus* (*Betacoronavirus* 1) within the genus *Betacoronavirus*. It is one of the four genera within the subfamily *Orthocoronavirinae* of the family *Coronaviridae* within the order *Nidovirales* [13, 14]. The genome size is about 31 kb and contains 10 open reading frames (ORFs) flanked by 5' and 3' untranslated regions transcribed into a nested set of 6–8 3′-coterminal subgenomic mRNAs encoding both structural and non-structural proteins [15–17].

Coronaviruses possess five structural and 16 nonstructural proteins (NSPs). Among the structural proteins, the N protein binds viral RNA, activates cellular activity and is encoded by ORF10. The M and E proteins contribute to envelope formation and initiate alpha-interferon activity, being encoded by ORF 9 and ORF 8, respectively [13, 17]. The HE and S proteins play key roles in binding to host cell receptors and are encoded by ORF 3 and ORF 4, respectively [10, 17]. For host cell fusion, the S protein, undergoes proteolytic cleavage into S1 and S2 subunits at the amino acid position 768–769 by cellular trypsin-like enzymes. The S1 subunit is located in the N-terminal region, containing the receptor-binding domain (RBD) responsible for binding to the host cell receptor. It plays a critical role in virus attachment, eliciting a neutralizing antibody response, and contributing to hemagglutinin activity.

The S1 subunit is commonly used in sequencing and phylogenetic analyses to identify mutations, circulating genotypes and variants [18–20]. The S2 subunit, located in the C-terminal region, facilitates virus fusion with the host cell [16, 18]. NSPs are predominantly produced during infection and are involved in proteolytic processes, genome replication and subgenomic mRNA synthesis (transcription). ORF1a encodes pp1a, containing nsp 1–11 s, while ORF1a and ORF1b together encode pp1ab, containing nsp1-16 [21, 22]. Although BCoV belongs to a single serotype, numerous antigenically distinct viruses circulate in the field [13, 23, 24]. These antigenic variations can pose challenges for protective measures such as vaccination [25].

BCoV is an interspecies transmissible virus with high mutation rates and adaptability [1, 2, 4, 5]. Beyond its significance in veterinary medicine, BCoV has garnered attention in the context of zoonotic diseases, particularly following the emergence of SARS-CoV-2. Neutralizing antibodies against SARS-CoV-2 have been detected in cattle [26], studies have shown that antibodies produced against BCoV in cattle milk recognize SARS-CoV-2 antigens [27]. Due to the conserved immune system features and genomic similarities among mammals, BCoV serves as a valuable model for studying human coronaviruses, shedding light on the zoonotic implications of coronavirus infections, particularly in the context of the SARS-CoV-2 pandemic [20, 27, 28].

BCoV presence has been detected across continents, including the Americas, Europe, Asia, Oceania, and Africa [20]. Particularly since 2010, it has been recognized as a prevalent pathogen causing diarrhea and respiratory diseases [10]. In an effort to control BCoV in Türkiye, pregnant cows are vaccinated to confer protection to calves through colostral antibodies. Depending on the specifications of the commercial vaccine, one or two doses are administered to pregnant cows prior to calving. However, the effectiveness of this vaccination strategy remains unclear at present. New vaccines and vaccination strategies need to be evaluated with inclusion of local strains circulating in Türkiye. Therefore, the aim of this study was the isolation, detection, investigation of the frequency and molecular characterization of circulating BCoVs in cattle farms located in the Thrace district (European border) of Türkiye. The study also aimed to determine potential genotypes having unique antigenic properties with higher prevalence to include in vaccine and vaccination strategies.

## Materials and methods

### Study population and sample collection

The farms from which the samples were collected were selected based on the voluntary participation of the farms to cover the cities in the Thrace district. A total of 47, including 12 integrated and 35 small-scale farms located in the Thrace district bordering the European Union and a farm in Uşak province in Türkiye, were visited between 2021 and 2022 (see Additional file 1). Visits were conducted from November through April with higher frequency during the cold season when the infection is more likely to be seen. A total of 281 samples, composed of 189 nasal/oropharyngeal and 92 fecal swabs were collected from calves showing diarrhea and respiratory signs. The farm location, age, sex and clinical signs were also recorded (Table 1).

**Table 1:** Farm locations, age groups, visit dates, sample types, vaccination status, and clinical signs of animals. ^a,b,c^

Date of visit	Farm location and type ^a^	Age groups	Vaccination status ^b^	Nasal/Oropharyngeal swabs (OS) ^c^	Fecal swabs (DS) ^c^	Clinical signs
13.01.2021	Tekirdağ/ Malkara (H.E./7)	0–6 month-old	UNV	18	1	Dyspnea, coughing, nasal discharge, weakness, diarrhea
14.01.2021	Kırklareli (E)	0–3 month-old	V	9	0	coughing, nasal discharge weakness
26.01.2021	Kırklareli (E)	0–6 month-old	V	24	16	Dyspnea, coughing, nasal discharge, weakness, diarrhea
05.02.2021	Kırklareli (E)	0–6 month-old	V	10	8	Diarrhea, coughing
10.02.2021	Edirne (H.E./9)	0–3 month-old	UNV	15	0	Coughing, nasal discharge, weakness
22.02.2021	Kırklareli /Lüleburgaz (E)	0–6 month-old	V	19	4	Dyspnea, coughing, nasal discharge, weakness, diarrhea
23.02.2021	Kırklareli (H.E./1)	0–12 month-old	UNV	1	2	Dyspnea, coughing, nasal discharge, weakness, diarrhea
24.02.2021	Kırklareli (E)	0–6 month-old	V	6	7	Dyspnea, coughing, nasal discharge, weakness, diarrhea
09.03.2021	Kırklareli (E)	0–6 month-old	V	4	10	Dyspnea, coughing, nasal discharge, weakness, diarrhea
18.03.2021	Kırklareli (E)	0–6 month-old	V	9	1	Dyspnea, coughing, nasal discharge, weakness
02.04.2021	Kırklareli (E)	0–6 month-old	V	5	17	Dyspnea, coughing, nasal discharge, weakness
07.04.2021	İstanbul/ Çilingirköy (H.E./2)	0–12 month-old	UNV	7	0	Fever, coughing
19.04.2021	Edirne (H.E./7)	0–12 month-old	UNV	12	0	Coughing, nasal discharge, weakness
04.11.2021	Edirne (H.E./3)	0–6 month-old	UNV	6	1	Coughing, nasal discharge, weakness
15.12.2021	Kırklareli (E)	0–3 month-old	U	0	15	Diarrhea
17.12.2021	Edirne (H.E./6)	0–6 month-old	UNV	11	0	Coughing, nasal discharge, weakness
05.01.2022	Uşak (E)	0–6 month-old	V	3	0	Coughing, nasal discharge, weakness
04.03.2022	Uşak (E)		V	20	0	Coughing, nasal discharge, weakness
07.03.2022	Kırklareli (E)	0–3 month-old	V	10	10	Fever, nasal discharge, weakness, diarrhea
Total	5 City			189	92	

^a^*E* Integrated farms; *HE* Small-scale farms/number of small-scale farms visited,

^b^*V* Vaccinated; *UNV* Non-vaccinated,

^c^*OS* Nasal/Oropharyngeal Swab; *DS* Fecal Swab

The date of visit was used as the farm code (E.g. 13.01.2021 is stated as a farm code 1301)

Duplicate samples were taken from each animal. Swabs intended for molecular analyses were preserved in 500 µl RNAlater solution (İnvitrogen, Cat. No: AM7021) while swabs intended for virus isolation were placed in 500 µl MEM (Capricorn Scientific, Cat. No: MEM-XA) containing 1% antibiotic (Penicillin–Streptomycin (10,000 U/mL) (Gibco™ Cat. No: 15140122). Samples were transported to the laboratory at a cold temperature (4–8 °C). All pregnant cows in the integrated farms were vaccinated against BCoV killed vaccine during gestation whereas none of the cows in the small-scale farms had implemented a BCoV vaccination protocol (Table 1).

### RNA extraction and cDNA synthesis

Viral RNA was extracted from fecal and nasal/oropharyngeal swabs, and cell culture supernatants were obtained during virus isolation studies using the PureLink^™^ RNA Mini Kit (Invitrogen, Cat. No: 12183018 A) as described by the manufacturer. Complementary DNA (cDNA) was synthesized using the High-Capacity cDNA reverse transcription kit (Invitrogen ^®^ Cat. No: 4368814) following the manufacturer's protocol (ThermoFisher Scientific, USA).

### SYBR-green real-time RT-PCR

To detect BCoV-RNA in samples, real-time RT-PCR was performed using *in-house* SYBR Green real-time RT-PCR assay with primers targeting the S1 region of the virus as the SYBR-based approach offers high analytical sensitivity. In addition, TaqMan probe-based primers from Decaro et al. (2008) targeting the M gene of BCoV were used to detect BCoV in cell culture supernatants and confirm viral replication of positive isolates [29]. Positive and negative controls were included for all PCR reactions. The BCoV positive control was obtained from samples previously identified as positive through RT-PCR and sequencing studies at the Department of Virology, Veterinary Faculty of Istanbul-Cerrahpasa, Istanbul, Türkiye whereas nuclease-free water was used for the negative control in place of the DNA template. Detailed information on the primers, master mixes and reaction conditions is provided in Table 2.

**Table 2:** Primers and PCR conditions

PCR	Target gene	Oligonucleotides (5’−3’)	Product size (bp)	Reaction mixture	Thermal cycle conditions	Reference
BCoV RT-PCR	S1	SP1F (10 pmol/µl): CTTATAAGTGCCCCCAAACTAAAT SP1R (10 pmol/µl): CCTACTGTGAGATCACATGTTTG	622	Master Mix (Thermo Scientific, Cat. No: K107): 12.5 µl SP1F: 1.50 µl SP1R: 1.50 µl DMSO: 1.25 µl Nuclease Free Water: 5.3 µl cDNA:3 µl	Initial Denaturation: 95 °C-3 min	Decaro et. al., 2008
					40 cycles of Denaturation: 95 °C-30 s Annealing: 51 °C-30 s Extension: 72 °C-30 s	
					Final Extension: 72 °C-5 min	
BCoV real-time RT-PCR SYBR Green	S1	895-F (10 pmol/µl): GCTCCCACAAATTTCTGTCCG 999-R (10 pmol/µl): AGGACAAGTGCCTATACCACT	105	Master Mix (Thermo Scientific, Cat. No: K0232): 12.5 µl 895-F:1 µl 999-R: 1 µl SYBR-Green:0.5 µl Nuclease Free Water: 7 µl cDNA: 3 µl	Initial Denaturation: 95 °C-10 min	In-House Primers 2014
					40 cycles of Denaturation: 95 °C-15 s Annealing: 60 °C-30 s 60 °C-1 min Melt Curve 60 °C-95°C, + 0,3 °C	
BCoV real-time RT-PCR TaqMan Probe	M	BCoV-F: (10 pmol/µl): CTGGAAGTTGGTGGAGTT BCoV-R: (10 pmol/µl): ATTATCGGCCTAACATACATC BCoV-Pb: (10 pMol/µl): FAMd-CCTTCATATCTATACACATCAAGTTGTT-BHQ1e	85	Master Mix (Thermo Scientific, Cat. No: K0232): 12.5 µl BCoV-F: 0.6 µl BCoV-R: 0.6 µl Probe: 0.2 µl Nuclease Free Water: 7 µl cDNA: 5 µl	Initial Denaturation: 95 °C-10 min 40 cycles of	Decaro et al., 2008
					Denaturation: 95 °C-15 s Annealing: 60 °C-30 s	

### Statistical evaluation

The Chi-Square statistical test was used to examine the relationship between the vaccinated and non-vaccinated status of samples identified as positive by real-time RT-PCR (Additional file 5).

### RT-PCR and sequence analysis

Sequence analysis was conducted using primers designed by Decaro et al. (2008) targeting the highly variable S1 region of BCoV spike gene, which plays a key role in virus-host interaction. A 622 bp segment from samples identified as positive by SYBR Green real-time RT-PCR (Table 2). The RT-PCR products were visualized using 1.5% agarose gel electrophoresis [29]. The products, where bands were observed on agarose gel within the expected size (622), were sent for the Sanger sequencing to a commercial company (MedSanTek Ltd, Istanbul, Türkiye).

Fourteen BCoV partial S1 sequences obtained, including a previously confirmed positive control re-sequenced for inclusion in the analysis, were submitted to NCBI GenBank. Their accession numbers are listed in Table 3.

**Table 3:** GenBank accession numbers and sample information for 14 BCoV partial S1 sequences

Number of used BCoVs strain	BCoV Partial S1 sequences collected in this study	GenBank accession number	Strain names (sample code/isolate source/province)
1	Türkiye	OQ831428	BCoV D Dec9
2	Usak	OQ821701	BCoV 403 2 OS Usak partial S1 gene
3	Istanbul	OQ831427	BCoV 704 2 OS Istanbul
4	Tekirdag	OQ821703	BCoV 1301 8 OS Tekirdag
5	Edirne	OQ821702	BCoV 1002 15 OS Edirne
6	Edirne	OQ821710	BCoV 1002 9 OS Edirne
7	Kirklareli	OQ821709	BCoV 2601 17 DS Kirklareli
8	Kirklareli	OQ821711	BCoV 2601 30 DS Kirklareli
9	Kirklareli	OQ821708	BCoV 2402 11 DS Kirklareli
10	Kirklareli	OQ821707	BCoV 2402 11 OS Kirklareli
11	Kirklareli	OQ821706	BCoV 2402 1 DS Kirklareli
12	Edirne	OQ821704	BCoV 1712 7 OS Edirne
13	Kirklareli	OQ821700	BCoV 703 12 OS Kirklareli
14	Kirklareli	OQ821705	BCoV 2202 4 OS Kirklareli

### Phylogenetic and heatmap analysis

Fourteen BCoV partial S1 sequences were obtained in this study (Table 3), other BCoVs (N = 20), four vaccine strains (N = 4), and the reference Mebus strain (N = 1), (in total, N = 25, see Additional file 2), coronaviruses (CoVs) of other animal species as well as human coronaviruses (N = 18, see Additional file 3) submitted to NCBI GenBank from different countries were used in the phylogenetic analysis. The selection criteria for the viruses to include in phylogenetic tree was targeted gene, geographic diversity and the relevance to viruses detected in this study. A total of 57 sequences including BCoV S1 and other CoVs were edited using Chromas Pro and aligned with MAFFT version 7 (online version) [30].

The Maximum Likelihood: The RAxML method was used to construct the phylogenetic tree with 1000 Bootstrap replicates by using MegAlign Pro Software (DNASTAR). GenBank accession numbers of the gene sequences used are provided in Additional File 2 of BCoVs and Additional File 3 of other CoVs.

### Virus isolation

To isolate BCoV in HRT-18 cell lines (provided by Dr Mariette Ducatez, INRA Toulouse, France), the method was validated by isolation study with positive control using trypsin. Nasal/oropharyngeal and fecal swabs brought to the laboratory in transport medium for isolation were vortexed (IKA Vortex Genius) with 1–2 mL MEM (Capricorn Scientific, Cat. No: MEM-XA), centrifuged at 3000 rpm for 10 min, then passed through a 0.22 μm filter. 1% antibiotic/antimycotic (Gibco, Cat. No: 15240062) solution was added and prepared for inoculation. HRT-18 cells were grown in Dulbecco's Modified Eagle Medium (DMEM, Gibco™ Cat. No: 21969035), supplemented with 10% fetal calf serum (FCS, Gibco^™^ Cat. No: 16000044) and 1% antibiotics (Penicillin–Streptomycin (10,000 U/mL) (Gibco^™^ Cat. No: 15140122). The cells were passaged into 25 cm^2^ flasks at a density of 700.000 cells per mL then into 6-well plates as 300.000 cells per mL and incubated in 5% CO_2_ incubator at 37 °C until they were 80–100% confluence. After removing the cell growth medium, cell surfaces were washed twice with DPBS (no calcium, no magnesium), using 2 mL for 25 cm^2^ flasks and 500 µl for each well of 6-well plates. To minimize possible cell overgrowth and the potential for serum-mediated trypsin inhibition during virus propagation, we used MEM without FBS for infection medium. Cells were treated with infection medium (MEM + 1% antibiotic/antimycotic containing 0.5 μl/mL of 0.25% trypsin–EDTA (Gibco^™^ Cat. No: 25200056) and incubated in a 5% CO_2_ incubator set at 37 °C for 3 h. After removal of the infection medium, 300 μl of inoculum was added to each well of the 6-well plates and 1 mL of inoculum was added to 25 cm^2^ flasks, with intermittent rocking for 1.5 h. After the incubation period, the inoculums were collected without damaging the bottom. To each well of the 6-well plate and the 25 cm^2^ flasks, 3 mL and 10 mL of infection medium (MEM + 1% antibiotic + 1 µl/mL trypsin) were added, respectively and placed in a 5% CO_2_ incubator set at 37 °C [31]. The cells were observed daily for the presence of cytopathic effect (CPE) for 7 days. At the end of 7 days, blind passage was performed with inoculums in which no CPE was observed but where the presence of virus was suspected. A maximum of 3 blind passages were planned. Inoculums in which CPE was observed passaged to increase the viral load and at each passage, cell supernatants were collected and probe-based real-time RT-PCR was performed to monitor the decrease in Threshold Cycles values (Ct).

## Results

### Clinical findings

**Fig. 1:**
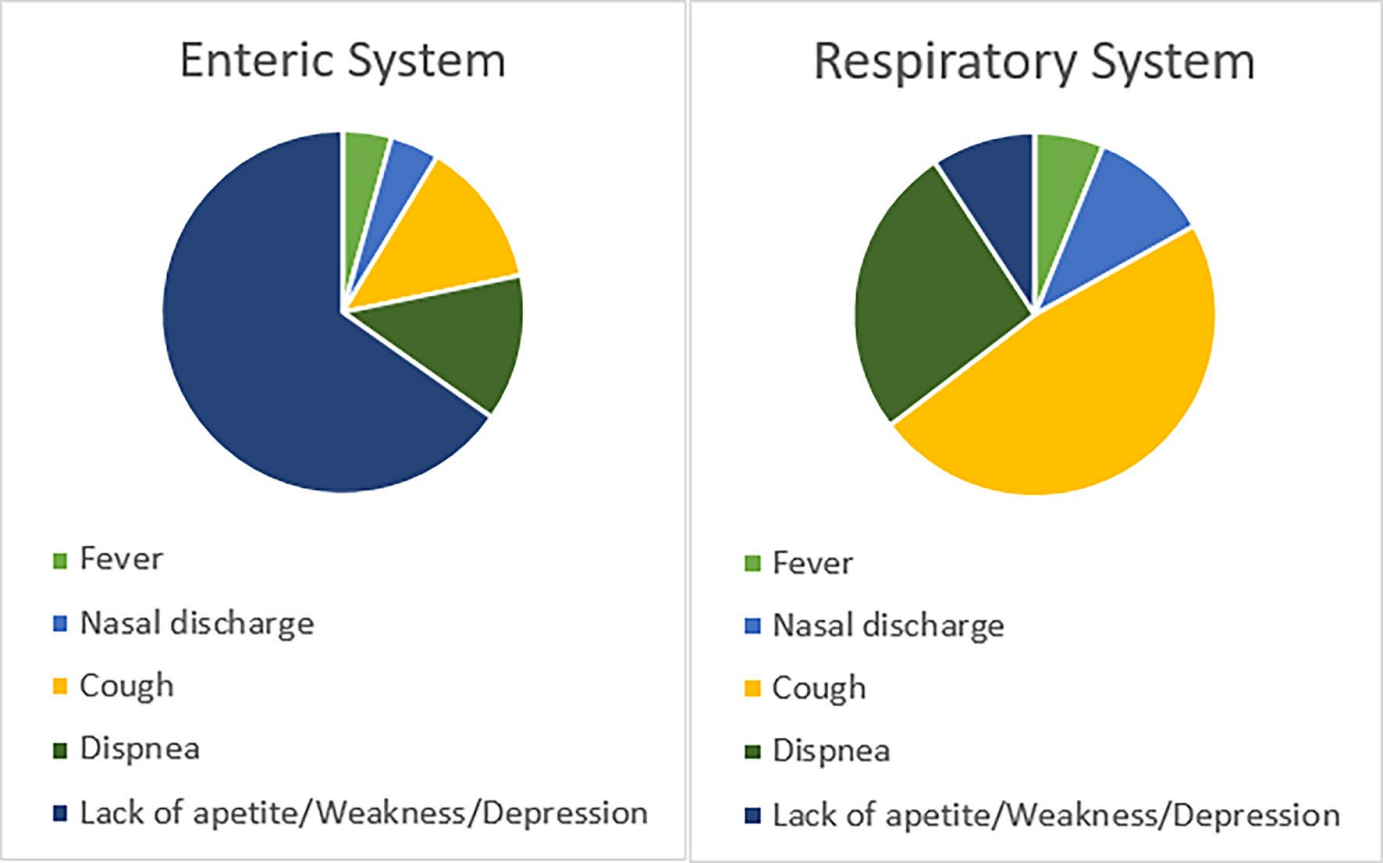
Graphs showing the proportion of signs associated with the enteric and respiratory systems that were found to be positive for BCoV

In addition to diarrhea and respiratory system disorders, one or more of the following signs were observed in the sampled animals: fever, anorexia, weakness, depression, serous eye discharge and seromucous nasal discharge. Diarrhea and respiratory signs were observed together in 6 of the sampled animals. The frequency of signs accompanied by diarrhea and respiratory system disorders in BCoV positive animals is given in Fig. 1.

### Detection of BCoV-RNA

#### Overall detection of BCoV by SYBR-Green real-time RT-PCR

For the real-time RT-PCR assay efficiency, the Ct were as follows: 21.68- 24.93- 28.22–31.07, respectively, for dilutions of the sample used as positive control prepared up to 10^3^ from the stock cDNA.

BCoV RNA was detected in 62 (62/281, 22.06%) of the total 281 swabs, of which 41 (41/189, 21.69%) were nasal/oropharyngeal swabs and 21 (21/92, 22.82%) were fecal swabs. BCoV RNA was detected in 45 of the 47 (95.74%) farms sampled. Of the farms where BCoV was not detected, one was an integrated farm (vaccinated farm, (code/sampling date): 204/02.04.2021, number of samples collected: 20) and the other was a small-scale farm (nonvaccinated farm, (code/sampling date): 2302/23.02.2021, number of samples: 3). BCoV RNA was detected in one or more samples in 11 out of the 12 integrated farms that were sampled (91.66%). BCoV RNA was also detected in one or more samples in 34 out of the 35 small-scale farms that were sampled (97.14%). Ct values for positive control and positive test samples were in the range of 12.57–40.9 (see Additional file 4). No Ct was observed in the negative control.

### The age status of BCoV-positive animals

Calves positive for BCoV-RNA by real-time RT-PCR were grouped according to their ages as 0–7 days, 8–30 days, 31–60 days and 61–180 days old (Table 4). In animals with diarrhea, the most common age range in which clinical signs were observed was 8–30 (56.2%, 52/92) days old. In these animals BCoV-RNA was most frequently detected in those aged 8–30 (28.84%, 15/52) days old. The age range in which the most common respiratory signs were observed was 61–180 (61.37%, 116/189) days old. In these animals, BCoV-RNA was most frequently detected in those aged 0–7 (40.00%, 2/5) days old.

**Table 4:** BCoV-RNA detection rates by age

BCoV detection rates according to age status	0–7 days old (positive/total sample)	8–30 days old (positive/total sample)	31–60 days old (positive/total sample)	61–180 days old (positive/total sample)	Total (positive/total sample)
Total number of BCoV detected swabs	3/17 (17.64%)	21/71 (29.57%)	14/72 (19.44%)	24/121 (19.83%)	62/281 (22.06%)
Number of BCoV detected fecal swabs	1/12 (8.33%)	15/52 (28.84%)	5/23 (21.73%)	0/5 (0%)	21/92 (22.82%)
Number of BCoV detected nasal/oropharyngeal swabs	2/5 (40%)	6/19 (31.57%)	9/49 (18.36%)	24/116 (20.68%)	41/189 (21.69%)

From birth to 1 month of age, the rate of BCoV detected in the respiratory system was 33.33% (8/24) and 25.00% (16/64) in the enteric system (Table 4).

### BCoV detection rates in vaccinated and non-vaccinated animals

**Fig. 2:**
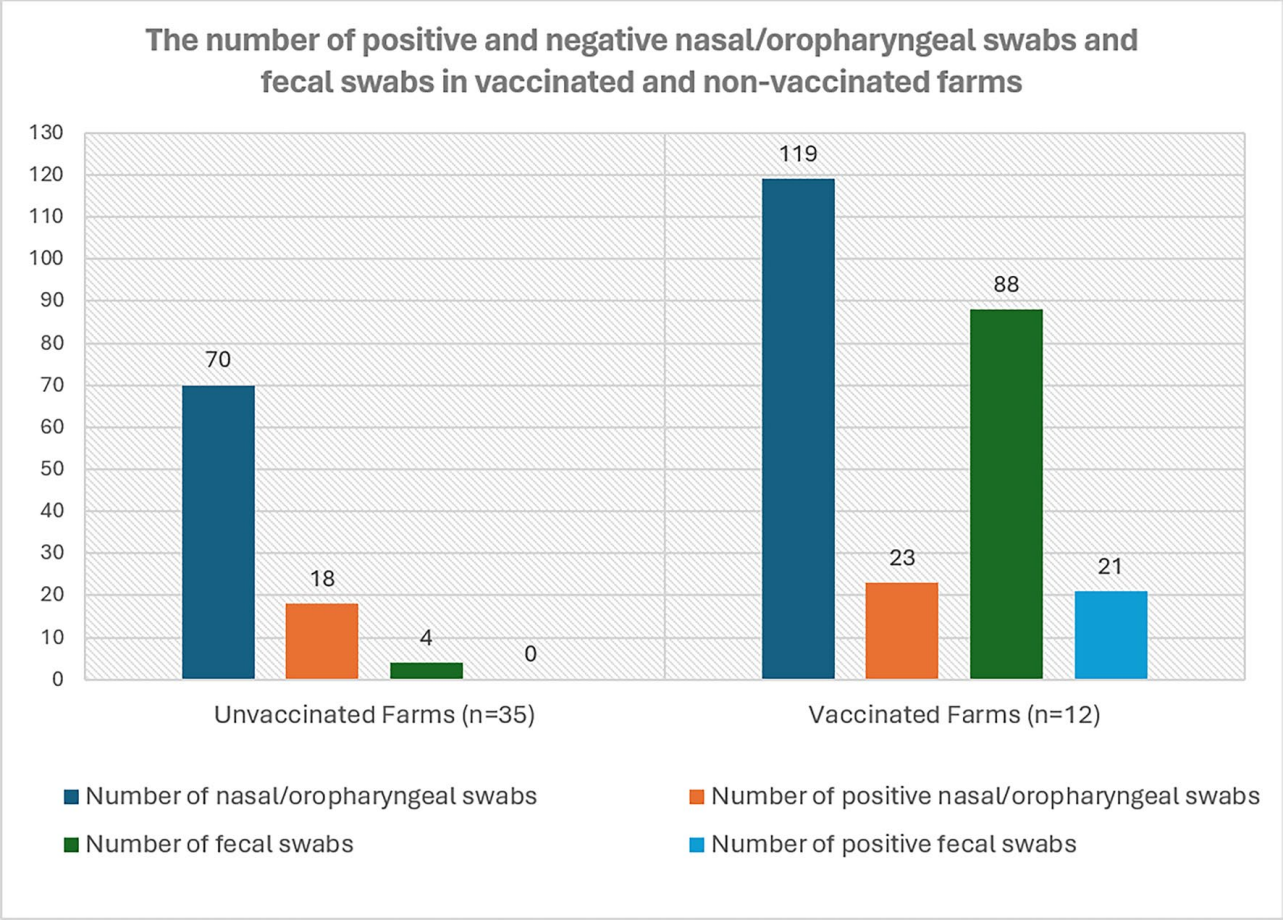
Histogram shows the number of positive and negative nasal/oropharyngeal swabs and fecal swabs in vaccinated and non-vaccinated farms

The number of positive and negative nasal/oropharyngeal swabs and fecal swabs in vaccinated and non-vaccinated farms is provided with graphical representation in Fig. 2. In vaccinated farms, dams were vaccinated with inactivated-combined (BCoV, Bovine Rotavirus, *E.coli*) vaccines for calf diarrhea approved for use in Türkiye. In total, BCoV-RNA was detected in 44 of 207 animals from vaccinated farms and in 18 of 74 animals from non-vaccinated farms (see Additional file 5).

No significant relationship was found in the statistical analysis of the association between the vaccinated and non-vaccinated animals found BCoV positive by real-time RT-PCR using the chi-square test (see Additional file 5). The Chi-Square statistic is quite low, indicating a weak relationship between the two variables. The P-value (0.702) is much higher than the typical threshold of 0.05. This indicates that there is no statistically significant difference in the distribution of positive and negative PCR test results between vaccinated and non-vaccinated farms. The expected frequencies are close to the observed frequencies, which is consistent with the finding that there is no relationship. This means that in this dataset, vaccination status did not significantly affect PCR test results.

Among farms with at least one real-time RT-PCR-positive sample (i.e., where BCoV is present), vaccinated farms had 44/185 (23.78%) positives and non-vaccinated farms had 18/71 (25.35%). Farms with zero positives (one unvaccinated, N = 3; one vaccinated, N = 22) were excluded (see Additional file 6).

### Seasonal variation in BCoV detection rates

PCR positivity was 26% (44/169) in winter (December, January, February) and 16.2% (17/105) in spring (March, April, May). Pearson’s chi-square was suggestive but not statistically significant (χ^2^ = 3.63, df = 1, p = 0.0568), with a similar finding on Fisher’s exact test (two-sided p = 0.073). Autumn (November) was 1/7 (14.3%) and is presented descriptively.

### Sequencing, phylogenetic and heatmap analysis

A 622-bp fragment of the BCoV S1 gene, amplified by RT-PCR from 13 field isolates, was sequenced and used for phylogenetic analysis (see Additional file 7).

**Fig. 3:**
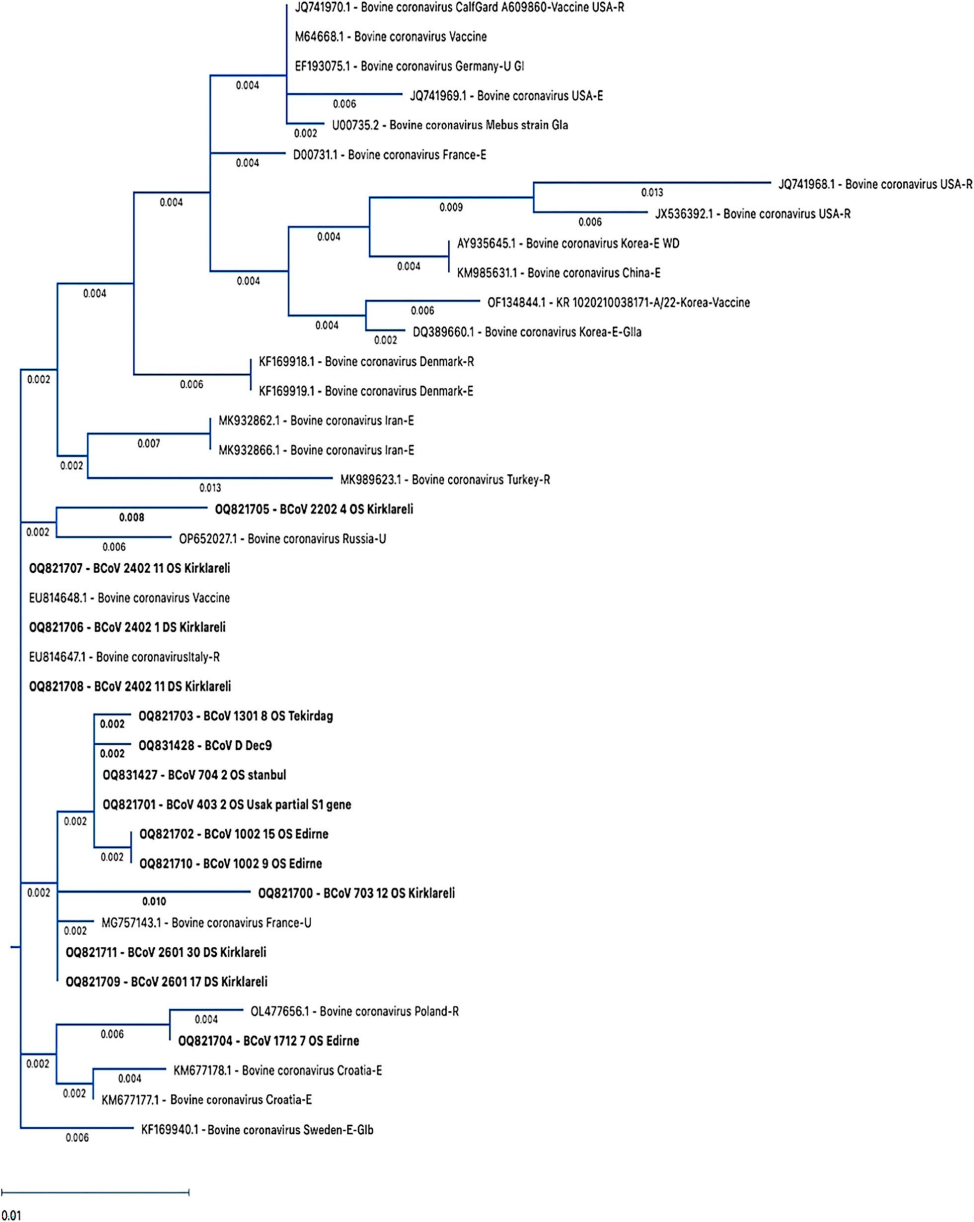
Phylogenetic tree constructed using partial S1 genome of BCoVs, including 25 reference BCoV strains from other countries, 13 BCoV strains identified in this study and one positive control were included. The BCoV strains obtained in this study are indicated in bold characters. Name order of reference sequences: Accession number/isolate name/country/isolate source: respiratory = R, enteric = E, BCoV subtype: GIa, GIb and GIIa [20]

In order to identify the genotypes and percentage similarity amongst viruses, the phylogenetic and Heatmap analyses were performed. According to the phylogenetic tree, the BCoV field strains obtained in this study showed high proximity to European strains and clustered within the GIb subgroup (Fig. 3).

**Fig. 4:**
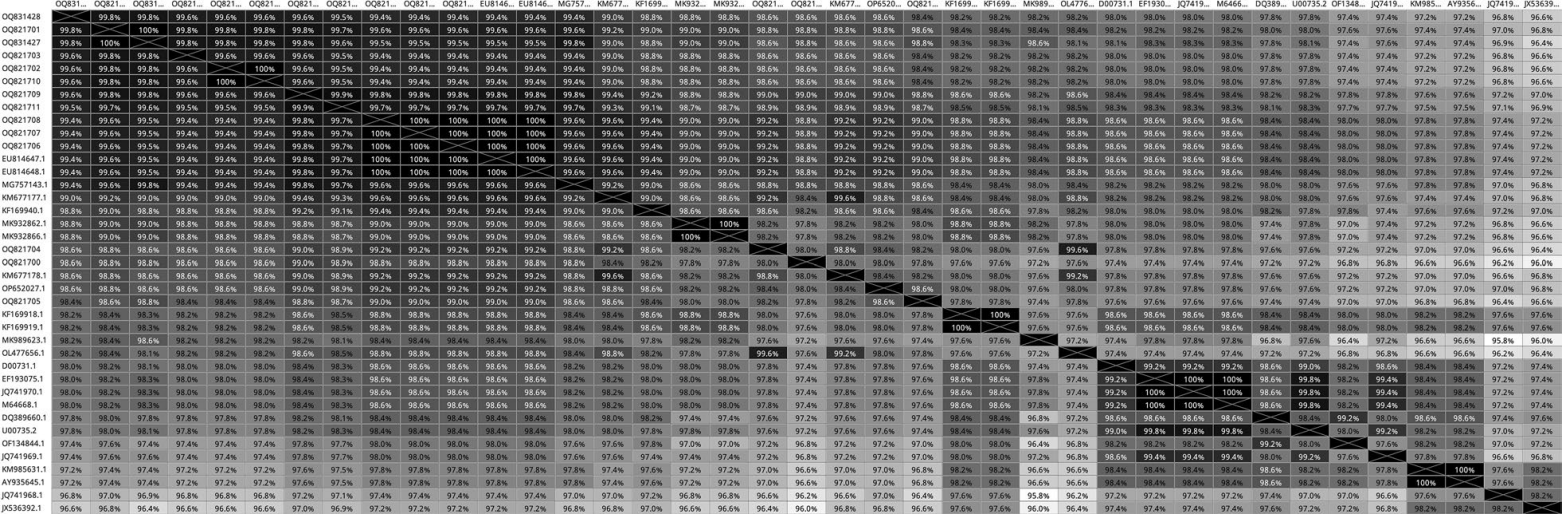
Heatmap analysis showing the similarity between BCoVs reference strains (N = 21), vaccine strains (N = 4) and BCoV strains detected in this study (N = 14)

Similarity rates between BCoVs circulating in the region (Thrace district and Usak province) ranged from 97.8% to 100% as indicated by the heatmap results. Samples 403–2 (Uşak/2022, OQ821701) and 704–2 (Istanbul/2021, OQ831427), were obtained from different regions and in different years, and were 100% identical and these two strains were the closest to the strain detected in France (MG757143.1, 99.8%), among the strains reported from other countries (Fig. 4, Additional file 8).

In this study, it was determined that the highest similarity between the field samples and other reported strains was observed with viruses detected in Italy (EU814647.1; 100%−98.8%), France (MG757143.1; 99.8–98.6%), and Poland (OL477656.1; 99.6%–97.6%). The lowest similarity was found with the US strains (JQ741968.1; 96.2%–97.4%, JX536392.1; 96%−97.2%). The similarity with the strains from neighboring Iran (MK932862.1, MK932866.1) ranged from 97.8% to 99% (Fig. 4, Additional file 8).

Among the vaccine strains included in the phylogenetic analysis based on the S1 gene sequences of the field strains the following similarities were observed: 98.8–100% with Bovine coronavirus Vaccine (EU814648.1; modified live vaccine strain), 97.4–98.6% with bovine coronavirus CalfGard A609860 vaccine USA-R (JQ741970.1; modified live vaccine strain), 97.4–98.6% with bovine coronavirus vaccine (M64668.1; BCV Norden vaccine strain), and 96.8–98% with KR 1020210038171-A/22-Korea vaccine (OF134844.1; BCV Inactive Group 1b vaccine strain). Our field samples exhibited a 97.2–98.4% similarity with the reference Mebus strain (Fig. 4, Additional file 8).

**Fig. 5:**
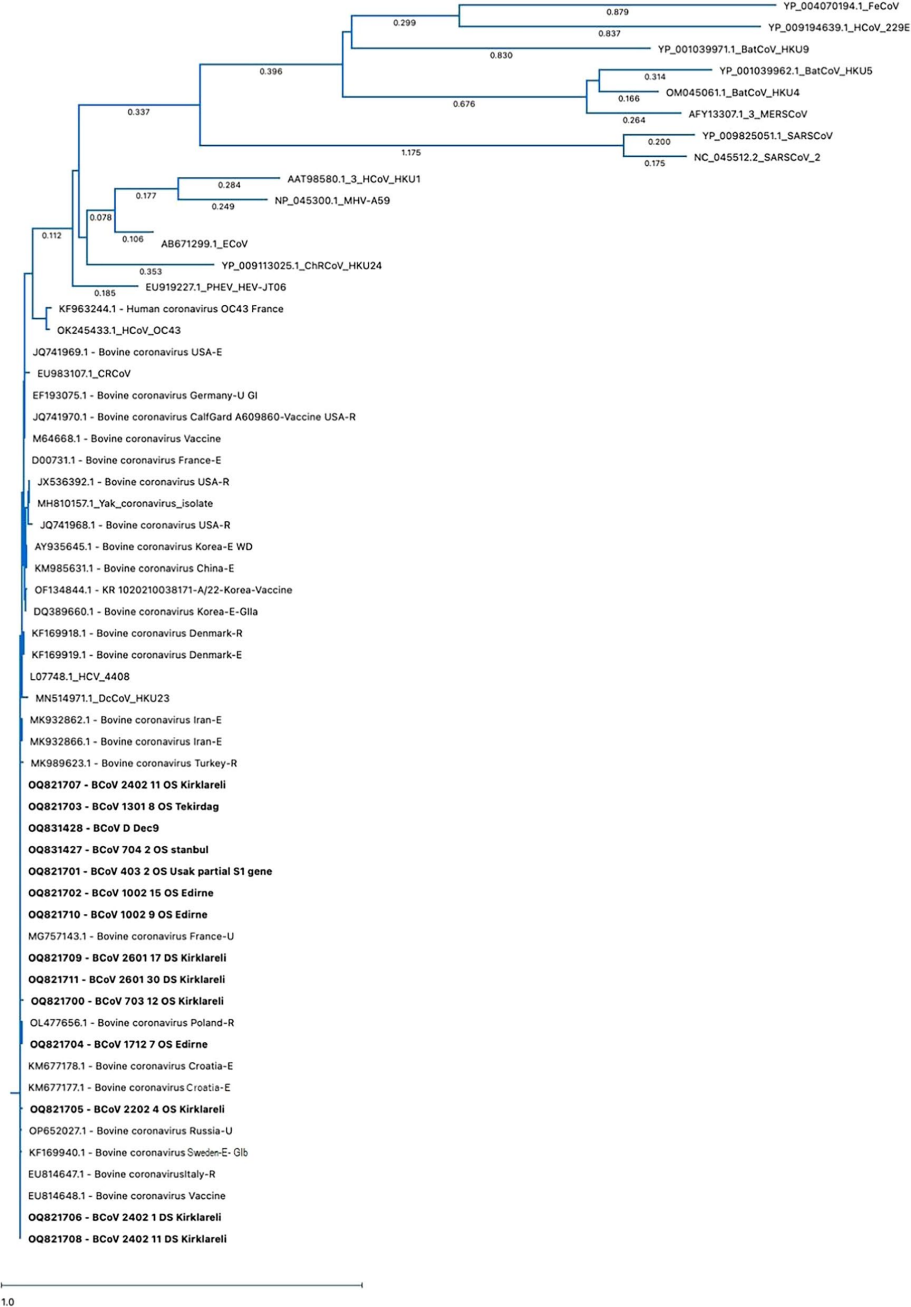
Phylogenetic tree constructed using reference coronavirus strains infecting animals and humans (N = 18), BCoVs from other countries (N = 25) and BCoV strains from this study (N = 14)

A second phylogenetic tree was constructed to analyze similarity between the bovine coronaviruses detected in this study and other animal/human CoVs (Fig. 5). According to the heatmap analysis, DcCoV_HKU23 (MN514971.1) was the most similar coronavirus among animal CoVs, with similarity rates of 97.85%−99.14%. Among human CoVs, the Human enteric coronavirus 4408 showed the highest similarity, with rates of 98.28%−99.14% to the field samples detected in this study (Table 5).

**Table 5:** Similarity rates by heatmap analysis between the other human and animal CoVs used in the phylogenetic analysis

GenBank accession numbers of other human and animal CoVs used for phylogenetic analysis	Similarity rates with the samples obtained from this study (%)	The most similar field sample in this study
PHEV_HEV-JT06	81.55–82.83	OQ821700
Yak_coronavirus_isolate	96.57–97.85	OQ821706, OQ821707, OQ821708
MHV-A59	70.82–72.10	OQ821703, OQ821702, OQ821701, OQ821710
DcCoV_HKU23	97.85–99.14	OQ831427, OQ821710, OQ821706, OQ821707, OQ821708, OQ821703, OQ821702, OQ821701
ECoV	79.83–80.69	OQ831427, OQ821710, OQ821703, OQ821702, OQ821701
CRCoV	97.42–97.85	OQ821704, OQ821709, OQ821711
ChRCoV_HKU24	74.24–75.11	OQ821701,
BatCoV_HKU9	48.50–49.79	OQ821704
BatCoV_HKU5	47.21–48.50	OQ821700
BatCoV_HKU4	51.07–51.50	OQ831427, OQ821710, OQ821711, OQ821709, OQ821703, OQ821702, OQ821701, OQ821700
FeCoV	42.92–43.35	OQ821700, OQ821704, OQ821706, OQ821707, OQ821708
SARSCoV	46.78–47.64	OQ831427, OQ821710, OQ821704 OQ821703, OQ821702, OQ821701, OQ821700
SARSCoV_2	46.35–47.64	OQ821705
HECV_4408	98.28–99.14	OQ821706, OQ821707, OQ821708x
HCoV_OC43	93.13–94.42	OQ821706, OQ821707, OQ821708
HCoV_HKU1	69.53–70.82	OQ831427, OQ821710, OQ821703, OQ821702, OQ821701, OQ821700
MERS-CoV	52.79–53.22	OQ831427, OQ821710, OQ821711, OQ821709, OQ821706, OQ821707, OQ821708, OQ821703, OQ821702, OQ821701,
HCoV_229E	45.92–46.35	OQ821708, OQ821707, OQ821706, OQ821704, OQ821700

### Cell culture and virus isolation

**Fig. 6:**
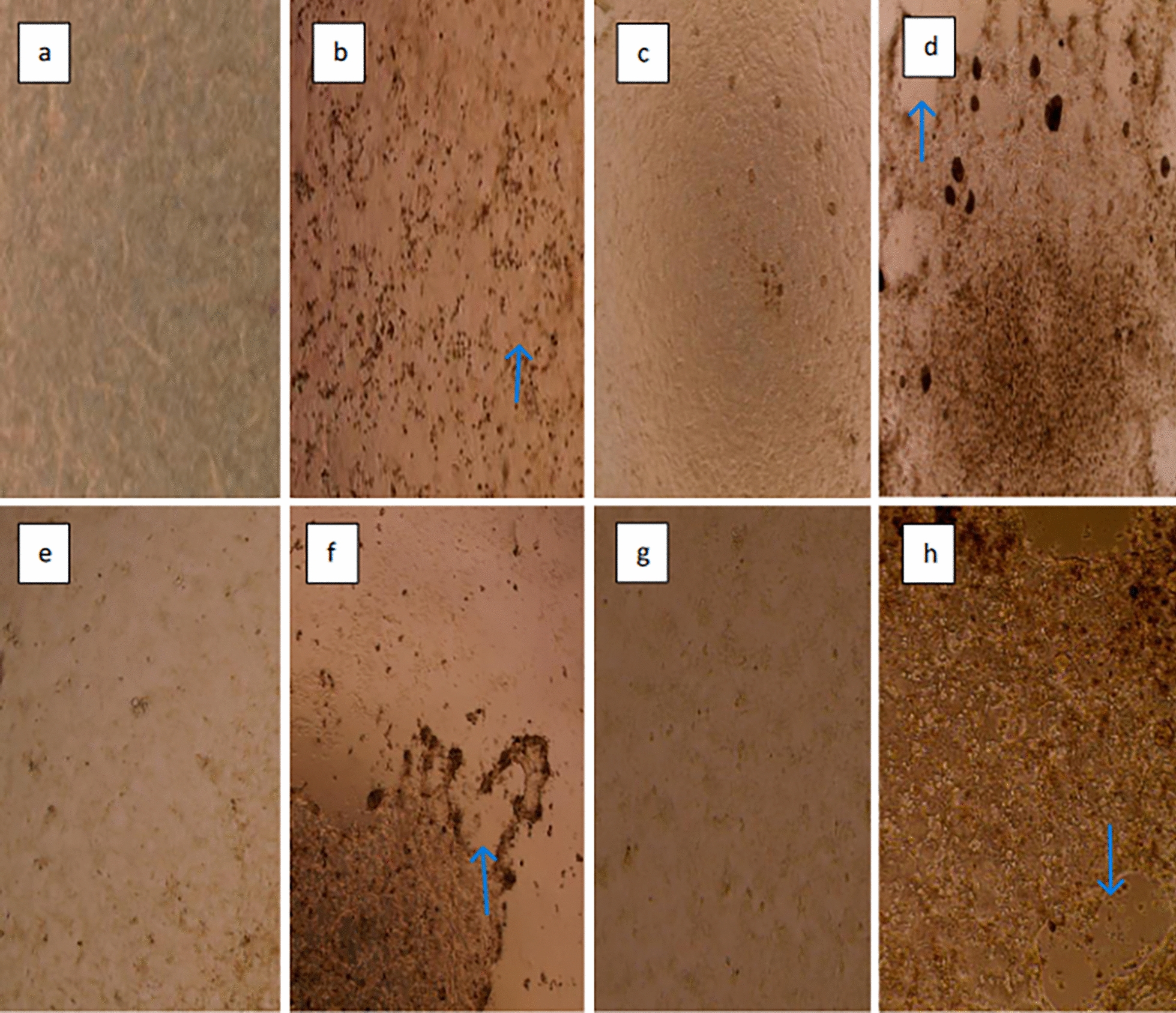
Isolation images of nasal/oropharyngeal swab (1002–6) sample using HRT-18 cells **a** control well used in the first inoculation/4X, **b** 1002–6 CPE image at first inoculation/4X, **c** 1 st passage control well/4X, **d** CPE image of the 1 st passage of 1002–6/4X, **e** 2nd passage control well/4X, **f** CPE image of the 2nd passage of 1002–6/4X, **g** 2nd passage control well/10X, **h** CPE image of the 2nd passage of 1002–6/10X (EVOS XL Core, Invitrogen). Detachment of the cells were marked with the blue arrows

**Fig. 7:**
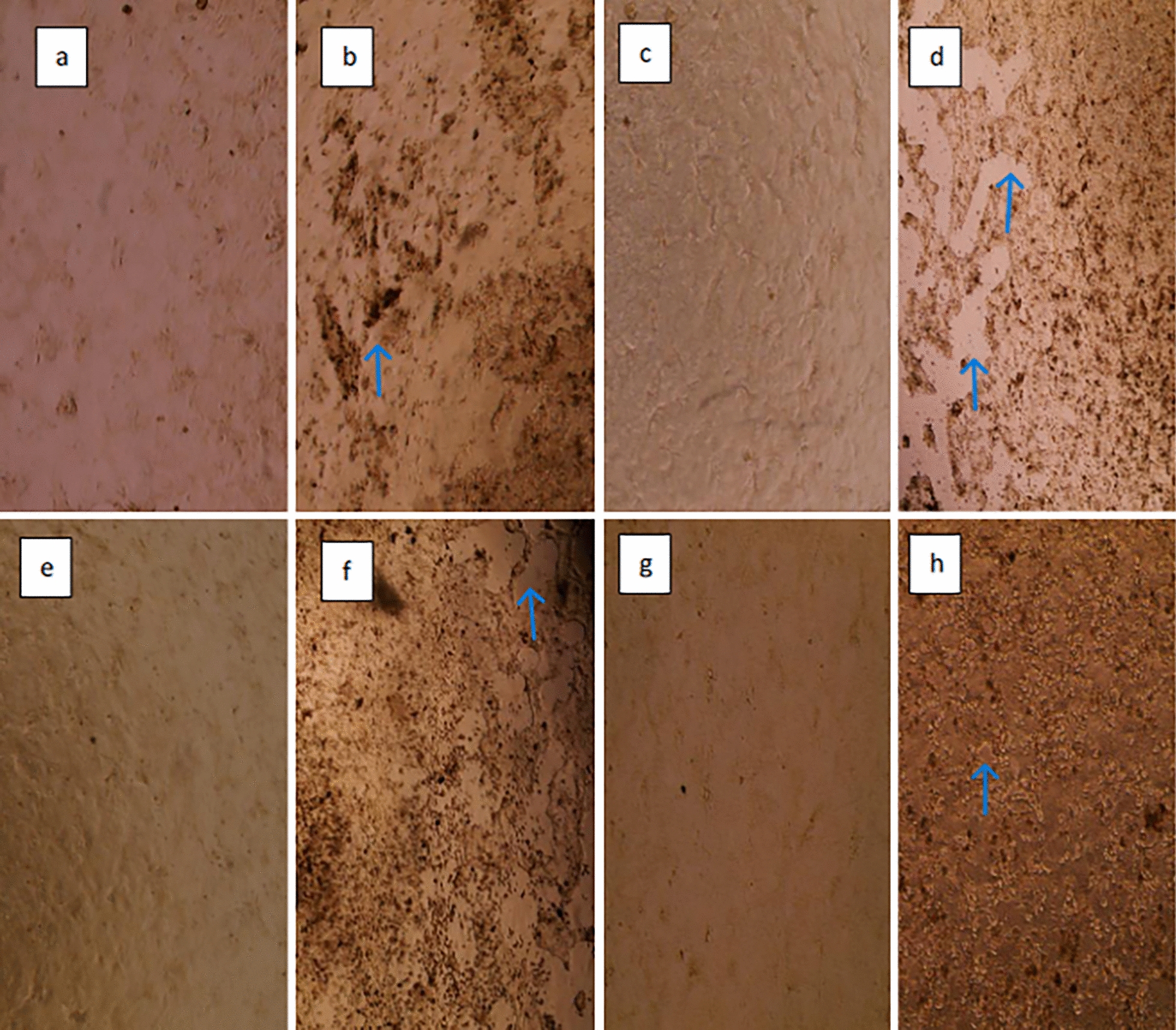
Isolation images of fecal swab (2601–17) sample using HRT-18 cells. **a** control well used in the first inoculation/4X, **b** 2601–17 CPE image at first inoculation/4X, **c** 1 st passage control well/4X, **d** CPE image of the 1 st passage of 2601–17/4X, **e** 2nd passage control well/4X, **f** CPE image of the 2nd passage of 2601–17/4X, **g** 2nd passage control well/10X, **h** CPE image of the 2nd passage of 2601–17/10X (EVOS XL Core, Invitrogen). Detachment of the cells were marked with the blue arrows

For virus isolation in cell cultures, samples with a low Ct value that tested positive real-time RT-PCR and RT-PCR were selected. BCoV was isolated from one fecal swab (2601–17, Ct: 18.74 by SYBR-Green real-time RT-PCR) and one nasal/oropharyngeal (1002–6, Ct: 13.66 by SYBR-Green real-time RT-PCR) swab by using HRT-18 cell lines. Cytopathic effects (CPEs) such as detachment of the cells (Figs. 6 and 7) were observed in the two field samples 48 h after the first inoculation. The percentage of detachment was 10% after 48 h of culture and about 70% after 5 days (Figs. 6 and 7). Cell supernatants were collected for re-passage and probe-based real-time RT-PCR was performed. As a result an amplification curve was observed at cycle 29.51 in fecal swab inoculum, and at cycle 17.27 for the nasal/oropharyngeal swab.

The viruses were re-passaged to increase the viral load, and CPEs were observed from the 48th hour onward during this second passage. Upon intensification of CPEs on the 5th day, the inoculums were collected, and real-time RT-PCR was performed on the cell supernatant. Probe-based real-time RT-PCR results showed a Ct value of 14.97 for the second passage of the nasal/oropharyngeal swab and 15.44 for the fecal swab. It was noted that there was a greater than 10^3^-fold decrease in Ct between the first and second passages.

Of the isolated samples, 2601–17 were sequenced and the information submitted to GenBank (GenBank accession number: OQ821709). The isolated viruses were passaged in cell culture three times including the first inoculation. The CPEs observed during isolation and the control cells are shown in Fig. 6 (1002–6), Fig. 7 (2601–17). No CPE was observed in the control cells and no Ct was observed in the control cells supernatant by probe-based real-time RT-PCR. Probe based real-time RT-PCR results for the cell supernatants are provided in Additional file 9.

## Discussion

BCoV infection affects cattle of all ages as well as wild ruminants, especially young cattle. It causes economic losses worldwide, including Türkiye due to digestive and respiratory disorders, mortality, and decrease in meat production [3, 4, 6–8, 13, 32]. Recent findings indicate that BCoV plays an important role in the occurrence of the BRDC [13]. Therefore, monitoring, molecular characterization of circulating strains and identifying new vaccine candidates are necessary to control the disease and establish vaccination strategies. Therefore, in this study, clinical findings, detection of coronaviruses, isolation, frequency, genotypes and variants of circulating coronaviruses (respiratory and enteric) in Thrace district were investigated. Since the S1 gene region of BCoV plays a major role in host cell recognition, binding, neutralizing antibody formation and immune response, we focused on the S1 gene region of BCoV for detection, partial sequencing and phylogenetic analysis in this study as used by other investigators [18, 19].

In the present study BCoV RNA was detected in 62 out 281 (22.06%) samples analyzed by SYBR-Green real-time RT-PCR. BCoV RNA was detected in one or more samples in 45 out of the 47 (95.74%) farms from which samples were collected. In a recent large-scale European study involving Belgium, Czech Republic, Denmark, France, Italy, Netherlands, Portugal, and Sweden, the molecular prevalence was reported to be 17%, with one or more animals testing positive in 50% of the sampled herds [33].

It has been reported that BCoV generally affects respiratory system of calves aged 2 weeks to 6 months [34]. A study conducted on 33 beef cattle farms in Sweden, focusing on BRD-associated viral pathogens, the molecular prevalence of BCoV was found to be 53.5%. Among calves up to 100 days old, the prevalence was more than twofold higher compared to those older than 100 days [34]. In this study, the molecular prevalence in respiratory system samples was 21.69% (41/189), with the detection rate in calves up to 1 month of age being 33% (8/24).

Clinical signs related to the respiratory system were most observed in calves aged 61–180 days, while the age range with the highest rate of BCoV positivity was 0–7 days. As demonstrated in the study by Alkheraif et al., this situation may be due to the synergistic effects of BRD pathogens suppressing the type 1 IFN response. Further studies involving other BRDC pathogens are needed to shed light on this issue [35].

BCoV RNA was detected in 22.82% of diarrheic calves in Thrace. Comparatively, a previous study conducted in the same district in 2016 reported a detection rate of 2% [36]. In this study, diarrhea was most common in calves aged 8–30 days (BCoV detected: 15 calves/calves with clinical signs at this age: 52/total fecal samples: 92), with the highest detection rate (28.84%) in this age group. The most common signs accompanying diarrhea in animals with positive fecal swabs were anorexia, weakness, and depression. For animals with positive respiratory samples, coughing and dyspnea were the most common signs.

The findings of this study show a higher detection rate of BCoV-RNA in the 8–30 days age group for both fecal swabs (28.84%) and nasal/oropharyngeal swabs (31.57%). This could be because of the decrease in maternally derived antibodies as well as the weak immune system of the young animals. On the other hand, the 0–7 days group has a high detection rate for nasal/oropharyngeal swabs (40%), but a much lower rate for fecal swabs (8.33%). Since we cannot determine the infection timeline in this observational study, a possible explanation could be earlier respiratory involvement with later intestinal shedding, consistent with prior reports that nasal shedding can precede fecal shedding in BCoV [13]. Further investigations may help to clarify this point.

In Türkiye, like in some other countries, dams are vaccinated with killed vaccine for BCoV during gestation and offspring are expected to be protected by ingested colostral antibodies. The rates of neonatal calf diarrhea in a previous study performed in Türkiye were reported to be 30% and 54% in calves born to vaccinated mothers and non-vaccinated dams, respectively [32]. It is stated that IgGs levels responsible for colostral immunity are 60 g/L in the first milking and decrease to 1 g/L in the 12th milking and are as much as 0.5 g/L in normal milk [37, 38]. Furthermore, it has been reported that BCoV frequency increased on days between 5–14, when colostral antibodies begin to fall [39]. In this study, BCoV was detected in animals in the first week of life (number of animals with clinical signs 17, number of BCoV detected 3).

Although colostral antibodies can protect animals for the first week of life, in this study, 3 animals infected with BCoV were from vaccinated farms, indicating that BCoV will be detected despite vaccination. In this study, no significant relationship was found between vaccination and positivity in the Chi-Square test in which the PCR positivity rate and vaccination status were classified. This data may suggest that inadequate colostral antibody transfer from the dam may have been an issue or insufficient antibody response to vaccination. It has been reported that even if neutralizing colostral antibodies are well established in mothers, calves may be reinfected with different BCoV strains [40]. Further studies are needed to investigate whether insufficient protection is due to the method of vaccine administration or the genotypic differences between the vaccine strain and the field strain and therefore the inability to produce neutralizing antibodies against the circulating virus. Mebus strain is generally used in the vaccines to immunize dams. The genotypic and antigenic mismatches between vaccine strain and circulating viruses is an important issue and warrants further investigations.

Phylogenetic and heatmap analyses in this study revealed high genotypic similarity (96.8–100%) between circulating BCoV strains in Thrace and vaccine strains. The highest similarity (98.8–100%) was observed with the modified live vaccine strain Bovine Coronavirus Vaccine (EU814648.1). Regional and temporal differences in BCoV genotypes have been documented, but samples from neighboring Thrace cities and European countries like Italy, France, and Poland showed high similarity (97.6–100%), as did samples from Iran (97.8–99%). Low similarity was observed with strains from the USA (JX536392.1, 96–97.2%) and Korea (AY935645.1, 96.6–97.8%), which are geographically distant from Türkiye. Zhu et al. (2022) reported close clustering between American and Asian strains (China, Vietnam, Japan) [20]. Kanno et al. (2007) and Kin et al. (2016) suggested that such close similarities might result from animal trade [24, 41]. This hypothesis could also explain the similarity observed between the samples in this study and the Iranian strains [42].

Suzuki and others (2020) reported that according to the phylogenetic analysis performed on whole genomes of BCoV isolates, the differences among BCoV tended to form clusters corresponding to the year of collection and/or the farm of origin rather than the disease type [17]. According to the phylogenetic and heatmap analyses of the present study, the findings support the evidence that the same field strain is circulating within a farm. For instance, the fecal swab samples 2601–17 (OQ821709) and 2601–30 (OQ821711), collected from different animals within the same farm, and the nasal/oropharyngeal swab sample 2402–11 (OQ821707) and fecal swab sample 2402–1 (OQ821706), also from different animals within the same farm, showed 100% similarity in the phylogenetic analysis of the S1 gene regions.

However, samples collected in different years (2022, Uşak province, accession number: OQ821701 and 2021, Edirne province, accession number: OQ831427) and different regions were also found to be 100% similar, which contrasts with the findings reported by Suzuki et al. [17]. The nucleotide similarity between viruses detected in Uşak province and Thrace district could be due to inner transport of animals inside Türkiye.

BCoV belongs to a single serotype, and although there is no clear distinction between enteric and respiratory coronaviruses, genotypic and virulence differences are thought to exist among strains circulating in the field [13, 20]. BCoVs are known to show dual tropism and it has been reported that the enteric isolate can mutate to resemble respiratory isolates [43]. In this study clinical signs of both systemic seen in one calf and according to phylogenetic analysis of the S1 region, enteric (2402–11, fecal swabs, OQ821708) and respiratory (2402–11, nasal/oropharyngeal swab, OQ821707) samples taken from the same calf were found to be 100% identical, indicating that there is no genotypic variation between the isolates from the respiratory and digestive systems.

BCoV belongs to the same genus as some human coronaviruses and causes similar symptoms (SARSC0V-2 and HECoV-4408). It also shows high nucleotide identity with some of them such as HECoV-4408 and HCoV-OC43. It has been stated that the protected structures of SARS-CoV-2 viral proteins, particularly M and S2, can be recognized by BCoV antibodies found in cow milk, which may provide complete or partial inactivation [27]. In a study, it was reported that neutralizing antibodies against the N and S protein of SARS-CoV-2 were detected in serum samples from cows on a farm where employees had contracted SARS-CoV-2, while the cattle tested negative for BCoV and other coronaviruses [26]. Such cases highlight the zoonotic importance of BCoV. Phylogenetic analyses of the S1 gene regions of other animal CoVs and human CoVs selected from GenBank along with the field strains obtained in this study, revealed that the highest similarity among human CoVs was with HECV-4408 (98,28–99.14%) and HCoV-OC43 (93.13–94.42%). Among other animal CoVs the highest similarity was observed with Dromedary Camel Coronavirus (DcCoV_HKU23; 97.85–99%; 14/MN514971.1) and Canine Respiratory Coronavirus (CRCoV; 97.42–97.85%; EU983107).

It has been reported that isolation of BCoV in cell culture is not always successful depending on cells and digestive enzymes [10]. The presence of digestive enzymes (trypsin, pancreatin, etc.) in the medium is recommended for the isolation of BCoV in HRT-18 cells [36, 44]. Isolation studies of BCoV obtained from the field showed that a trypsin-like digestive enzyme was required. It was also observed that the amount of trypsin used was critical and trypsin concentration higher than 1 µl/mL damaged the cells. A high viral load in the samples used for isolation was determined to be one of the most important factors for success. Additionally, maintaining the cold chain during the transport of samples to the laboratory was found to be crucial. In this study, the treatment of HRT-18 cells with a trypsin-containing infection medium prior to inoculation was found to be beneficial.

There are few limitations of this study. The number of samples collected for locality and age groups were different (e.g., 17 for 0–7 days age group and 121 for 61–180 days age group) because of difficulties during sample collection. This could effect the statistical significance and generalizability of findings for this age range. A bigger size of the S1 gene could have been targeted for sequencing but it was not performed because of some constraints of the study. The serological analyses could have been done in sampled animals to check antibody levels.

## Conclusion

This study shows that BCoV remains a significant health concern in calves in Türkiye and is clustered within the GIb subgroup alongside European strains.

Molecular prevalence rates underscore the widespread nature of the virus, and its detection in vaccinated farms raises concerns about vaccine efficacy. To better inform regional vaccine updates, collecting immunological data is also essential.

Although our 622-bp S-gene fragment is highly similar to HECV-4408 and HCoV-OC43, this is not sufficient to infer zoonotic potential. Integrated (One Health) surveillance, together with full-genome sequencing and epidemiological studies, will be required to assess cross-species risk.

The phylogenetic findings of this study highlight genetic similarities between strains from neighboring countries and distant regions, emphasizing the role of animal trade in viral dissemination.

Further research should focus on understanding the mechanisms of vaccine failure, the dynamics of colostral immunity, and the dual tropism of BCoV in respiratory and enteric systems.

Improved biosecurity measures, regular monitoring and the development of new vaccination strategies are necessary to control BCoV infections.

## Supplementary Information

Additional file 1. Title of data: Supplementary Figure S1. Description of data: Map of Türkiye showing the locations and sample numbers of the provinces where sampling was conducted

Additional file 2. Title of data: Supplementary Table S2. Description of data: BCoV sequences obtained from GenBank

Additional file 3. Title of data: Supplementary Table S3: Description of data: Metadata compiled for all human and animal CoV reference sequences used in this study, including host species and country of origin, viral genus/subgenus, virus name, and GenBank accession numbers

Additional file 4. Title of data: Supplementary Figure S4. Description of data: Ct and melting curves of samples detected positive by SYBR-Green real-time RT-PCR. The letters given alphabetically indicate the sample codes. a:502/14-OS, b:1002/10-OS, c:1002/12-OS, d:2202/4-OS, e:1002/15-OS, f: BCoV positive control, g: negative control

Additional file 5. Title of data: Supplementary Table S5. Description of data: Chi-square analysis of BCoV positivity in samples from vaccinated and unvaccinated farms

Additional file 6. Title of data: Supplementary Table S6. Description of data: Descriptive data for farms with ≥1 PCR-positive sample, showing nasal/oropharyngeal and fecal swab results in vaccinated and non-vaccinated farms

Additional file 7. Title of data: Supplementary Figure S7. Description of data: Representative agarose gel showing the expected 622-bp BCoV S1 amplicon, including positive and negative field samples and positive and negative controls. 1: Marker. 2: 2601/19; 3: Positive control; 4: Negative control; 5: 2601/25; 6: 1401/4; 7: 2601/30; 8: 2402/11; 9: 502/14. Materials and reagents used for gel preparation and visualisation: Agarose, İnvitrogen, Cat. No: 16500100; TAE Buffer, Thermo Scientific, Cat. No:15558042; Loading Dye 6X, Thermo Scientific, Cat. No: R0611; Nucleic acid stain, Biomatik, Cat. No: A4205, Marker, Biomatik, Cat. No:M7123-100Loads)

Additional file 8. Title of data: Supplementary Table S8: Description of data: Heatmap analysis table in Excel format for the 39 BCoV strains used in this study. BCoV field isolates obtained in this study and positive control N=14, Other BCoV sequences obtained from GenBank N=25

Additional file 9. Title of data: Supplementary Figure S9. Description of data: real-time RT-PCR test results of samples taken from cell supernatants after the first inoculation and 1 st passage of the thesis samples. a: Ct of the 1 st passage of 1002-6/14.97, b: Ct of the 1 st passage of 2601-17/15.44, c: Ct of the 1 st inoculum of 1002-6/17.27, d: PCR positive control/Ct: 25.68, e: Ct of the 1 st inoculum of 2601-17/29.51, f: PCR negative control/Ct: no Ct, g: virus isolation control well/Ct: no Ct

## Data Availability

All data are included in the manuscript. The data are available upon request from the corresponding author. Accession numbers are available in Figs. 3, 4 and 5. Sequences submitted to GenBank can be downloaded from NCBI.

## Abbreviations

CD: Calf diarrhea
WD: Winter dysentery
ORF: Open reading frame
RBD: Receptor binding domain
NSP: Nonstructural protein
MEM: Minimum essential medium
DMEM: Dulbecco’s modified eagle medium
cDNA: Complementary DNA
CPE: Cytopathic effect
NCBI: National center for biotechnology information
HRT-18: Human rectal tumor cell line-18
Ct: Threshold cycles value
BRD: Bovine respiratory disease
IgG: Immunoglobulin G

## References

[1] Zhang XM, Herbst W, Kousoulas KG, Storz J. Biological and genetic characterization of a hemagglutinating coronavirus isolated from a diarrhoeic child. J Med Virol. 1994;44(2):152–61. 7852955 10.1002/jmv.1890440207 PMC7166597

[2] Vijgen L, Keyaerts E, Lemey P, Maes P, Van Reeth K, Nauwynck H, et al. Evolutionary history of the closely related group 2 coronaviruses: porcine hemagglutinating encephalomyelitis virus, bovine coronavirus, and human coronavirus OC43. J Virol. 2006;80(14):7270–4. 16809333 10.1128/JVI.02675-05 PMC1489060

[3] Kaneshima T, Hohdatsu T, Hagino R, Hosoya S, Nojiri Y, Murata M, et al. The infectivity and pathogenicity of a group 2 bovine coronavirus in pups. J Vet Med Sci. 2007;69(3):301–3. 17409649 10.1292/jvms.69.301

[4] Amer HM. Bovine-like coronaviruses in domestic and wild ruminants. Anim Health Res Rev. 2018;19(2):113–24. 30683171 10.1017/S1466252318000117 PMC7108644

[5] Savard C, Provost C, Ariel O, Morin S, Fredrickson R, Gagnon CA, et al. First report and genomic characterization of a bovine-like coronavirus causing enteric infection in an odd-toed non-ruminant species (Indonesian tapir, *Acrocodia indica*) during an outbreak of winter dysentery in a zoo. Transbound Emerg Dis. 2022;69(5):3056–65. 34427399 10.1111/tbed.14300 PMC8943714

[6] Boileau MJ, Kapil S. Bovine coronavirus associated syndromes. Vet Clin North Am Food Anim Pract. 2010;26(1):123–46. 20117547 10.1016/j.cvfa.2009.10.003 PMC7125561

[7] Timurkan M, Aydın H, Belen S. Erzurum bölgesinde siğirlarda respiratory coronavirus enfeksiyonunun RT-PCR ile tespiti ve Moleküler Karakterizasyonu. Atatürk Üniversitesi Veteriner Bilimleri Dergisi. 2015;10:3.

[8] Yilmaz A, Umar S, Turan N, Kayar A, Richt JA, Yilmaz H. Current scenario of viral diseases and vaccination strategies of cattle in Turkey. J Infect Dev Ctries. 2022;16(8):1230–42. 36099365 10.3855/jidc.14767

[9] Mebus CA, White RG, Stair EL, Rhodes MB, Twiehaus MJ. Neonatal calf diarrhea: results of a field trial using a reo-like virus vaccine. Vet Med Small Anim Clin. 1972;67(2):173–217. 4334051

[10] Saif LJ. Bovine respiratory coronavirus. Vet Clin North Am Food Anim Pract. 2010;26(2):349–64. 20619189 10.1016/j.cvfa.2010.04.005 PMC4094360

[11] Saif LJ. Winter dysentery. Food Animal Pract. 2009. 10.1016/B978-141603591-6.10026-0.

[12] Toftaker I, Holmøy I, Nødtvedt A, Østerås O, Stokstad M. A cohort study of the effect of winter dysentery on herd-level milk production. J Dairy Sci. 2017;100(8):6483. 28601443 10.3168/jds.2017-12605 PMC7094253

[13] Vlasova AN, Saif LJ. Bovine coronavirus and the associated diseases. Front Vet Sci. 2021;8:643220. 33869323 10.3389/fvets.2021.643220 PMC8044316

[14] Woo PCY, de Groot RJ, Haagmans B, Lau SKP, Neuman BW, Perlman S, et al. ICTV virus taxonomy profile: coronaviridae 2023. J Gen Virol. 2023;104:4. 10.1099/jgv.0.001843 PMC12135074 37097842

[15] Cox GJ, Parker MD, Babiuk LA. Bovine coronavirus nonstructural protein ns2 is a phosphoprotein. Virology. 1991;185(1):509–12. 1833877 10.1016/0042-6822(91)90810-X PMC7131063

[16] Martínez N, Brandão PE, de Souza SP, Barrera M, Santana N, de Arce HD, et al. Molecular and phylogenetic analysis of bovine coronavirus based on the spike glycoprotein gene. Infect Genet Evol. 2012;12(8):1870–8. 22634277 10.1016/j.meegid.2012.05.007 PMC7106151

[17] Suzuki T, Otake Y, Uchimoto S, Hasebe A, Goto Y. Genomic characterization and phylogenetic classification of bovine coronaviruses through whole genome sequence analysis. Viruses. 2020;12(2):183. 32041103 10.3390/v12020183 PMC7077292

[18] Yoo D, Deregt D. A single amino acid change within antigenic domain II of the spike protein of bovine coronavirus confers resistance to virus neutralization. Clin Diagn Lab Immunol. 2001;8(2):297–302. 11238212 10.1128/CDLI.8.2.297-302.2001 PMC96053

[19] Decaro N, Campolo M, Mari V, Desario C, Colaianni ML, Di Trani L, et al. A candidate modified-live bovine coronavirus vaccine: safety and immunogenicity evaluation. New Microbiol. 2009;32(1):109–13. 19382676

[20] Zhu Q, Li B, Sun D. Advances in bovine coronavirus epidemiology. Viruses. 2022;14(5):1109. 35632850 10.3390/v14051109 PMC9147158

[21] Chen Y, Liu Q, Guo D. Emerging coronaviruses: genome structure, replication, and pathogenesis. J Med Virol. 2020;92(4):418–23. 31967327 10.1002/jmv.25681 PMC7167049

[22] Rohaim MA, El Naggar RF, Clayton E, Munir M. Structural and functional insights into non-structural proteins of coronaviruses. Microb Pathog. 2021;150:104641. 33242646 10.1016/j.micpath.2020.104641 PMC7682334

[23] Park SJ, Jeong C, Yoon SS, Choy HE, Saif LJ, Park SH, et al. Detection and characterization of bovine coronaviruses in fecal specimens of adult cattle with diarrhea during the warmer seasons. J Clin Microbiol. 2006;44(9):3178. 16954245 10.1128/JCM.02667-05 PMC1594715

[24] Kanno T, Hatama S, Ishihara R, Uchida I. Molecular analysis of the S glycoprotein gene of bovine coronaviruses isolated in Japan from 1999 to 2006. J Gen Virol. 2007;88(Pt 4):1218–24. 17374765 10.1099/vir.0.82635-0

[25] Fulton RW, Ridpath JF, Burge LJ. Bovine coronaviruses from the respiratory tract: antigenic and genetic diversity. Vaccine. 2013;31(6):886–92. 23246548 10.1016/j.vaccine.2012.12.006 PMC7115418

[26] Fiorito F, Iovane V, Pagnini U, Cerracchio C, Brandi S, Levante M, et al. First description of serological evidence for SARS-CoV-2 in lactating cows. Animals. 2022;12(11):1459. 35681922 10.3390/ani12111459 PMC9179237

[27] Arenas A, Borge C, Carbonero A, Garcia-Bocanegra I, Cano-Terriza D, Caballero J, et al. Bovine coronavirus immune milk against COVID-19. Front Immunol. 2021;12:637152. 33833758 10.3389/fimmu.2021.637152 PMC8021920

[28] Watanabe M, Ohnishi T, Arai S, Kawakami T, Hayashi K, Ohya K, et al. Survival of SARS-CoV-2 and bovine coronavirus on common surfaces of living environments. Sci Rep. 2022;12(1):10624. 35739204 10.1038/s41598-022-14552-9 PMC9218704

[29] Decaro N, Elia G, Campolo M, Desario C, Mari V, Radogna A, et al. Detection of bovine coronavirus using a TaqMan-based real-time RT-PCR assay. J Virol Methods. 2008;151(2):167–71. 18579223 10.1016/j.jviromet.2008.05.016 PMC7112840

[30] Kumar S, Stecher G, Tamura K. MEGA7: molecular evolutionary genetics analysis version 7.0 for bigger datasets. Mol Biol Evol. 2016;33(7):1870–4. 27004904 10.1093/molbev/msw054 PMC8210823

[31] Shin J, Choe S, Park GN, Song S, Kim KS, An BH, et al. Isolation and genetic characterization of a bovine coronavirus KBR-1 strain from calf feces in South Korea. Viruses. 2022;14(11):2376. 36366474 10.3390/v14112376 PMC9695762

[32] Alkan F, Burgu I, Can-şahna K, Çokçalışkan C. Yeni doğan buzağı ishallerine karşı ticari aşı ile aşılanan sığırlardan doğan yavrularda pasif bağışıklık düzeyi. Ankara Univ Vet Fak Derg. 2004;1(50):047–53.

[33] Berge AC, Vertenten G. Prevalence, biosecurity and risk management of bovine coronavirus infections on dairy farms in Europe. In Madrid: International Veterinary Information Service; 2022 4–8 Sept: 217–218.

[34] Studer E, Schönecker L, Meylan M, Stucki D, Dijkman R, Holwerda M, et al. Prevalence of BRD-related viral pathogens in the upper respiratory tract of Swiss veal calves. Animals. 2021;11(7):1940. 34209718 10.3390/ani11071940 PMC8300226

[35] Alkheraif AA, Topliff CL, Reddy J, Massilamany C, Donis RO, Meyers G, et al. Type 2 BVDV Npro suppresses IFN-1 pathway signaling in bovine cells and augments BRSV replication. Virology. 2017;507:123–34. 28432927 10.1016/j.virol.2017.04.015

[36] Pestil Z, Gülyaz V, Hasöksüz M. Marmara Bölgesinde Yeni Doğan Buzağı İshallerinde Bovine Coronavirusların Saptanması ve Patojenite Çalışması. Etlik Veteriner Mikrobiyoloji Dergisi. 2016;27(1):16–20.

[37] Erdoğan HM, Gökçe E. Neonatal Buzağılarda Kolostral İmmunoglobulinlerin Pasif Transferi Turkiye Klinikleri. J Vet Sci. 2013;4(1):18–46.

[38] Kozat S. Yenidoğan Buzağılarda Kolostrum Yönetiminin Önemi. Atatürk Üniversitesi Veteriner Bilimleri Dergisi. 2019;14(3):343–53.

[39] Crouch CF, Oliver S, Francis MJ. Serological, colostral and milk responses of cows vaccinated with a single dose of a combined vaccine against rotavirus, coronavirus and *Escherichia coli* F5 (K99). Vet Rec. 2001;149(4):105–8. 11504200 10.1136/vr.149.4.105

[40] El-Kanawati ZR, Tsunemitsu H, Smith DR, Saif LJ. Infection and cross-protection studies of winter dysentery and calf diarrhea bovine coronavirus strains in colostrum-deprived and gnotobiotic calves. Am J Vet Res. 1996;57(1):48–53. 8720237

[41] Kin N, Miszczak F, Diancourt L, Caro V, Moutou F, Vabret A, et al. Comparative molecular epidemiology of two closely related coronaviruses, bovine coronavirus (BCoV) and human coronavirus OC43 (HCoV-OC43), reveals a different evolutionary pattern. Infect Genet Evol. 2016;40:186–91. 26969241 10.1016/j.meegid.2016.03.006 PMC7106199

[42] Esmaeili H, Ghorani M, Hamidiya Z, Joghataei SM, Villanueva-Saz S, Lacasta D. Causes of abortion in Iranian goat herds and associated risk factors. Prev Vet Med. 2025;234:106381. 39536431 10.1016/j.prevetmed.2024.106381

[43] Zhang X, Hasoksuz M, Spiro D, Halpin R, Wang S, Vlasova A, et al. Quasispecies of bovine enteric and respiratory coronaviruses based on complete genome sequences and genetic changes after tissue culture adaptation. Virology. 2007;363(1):1–10. 17434558 10.1016/j.virol.2007.03.018 PMC7103286

[44] Benfield DA, Saif LJ. Cell culture propagation of a coronavirus isolated from cows with winter dysentery. J Clin Microbiol. 1990;28(6):1454–7. 10.1128/jcm.28.6.1454-1457.1990. 2166085 10.1128/jcm.28.6.1454-1457.1990 PMC267955

