# Supplementary material for: Molecular characterization of BCoV infecting vaccinated and non-vaccinated cattle in Thrace district Türkiye and isolation of field strains
Source: Virol J. 2025 Dec 1;22:388. doi: 10.1186/s12985-025-03010-3 (PMC12667072; doi:10.1186/s12985-025-03010-3)
Supplement: Supplementary file 1 — Additional file 1. Title of data: Supplementary Figure S1. Description of data: Map of Türkiye showing the locations and sample numbers of the provinces where sampling was conducted [file 12985_2025_3010_MOESM1_ESM.pdf]

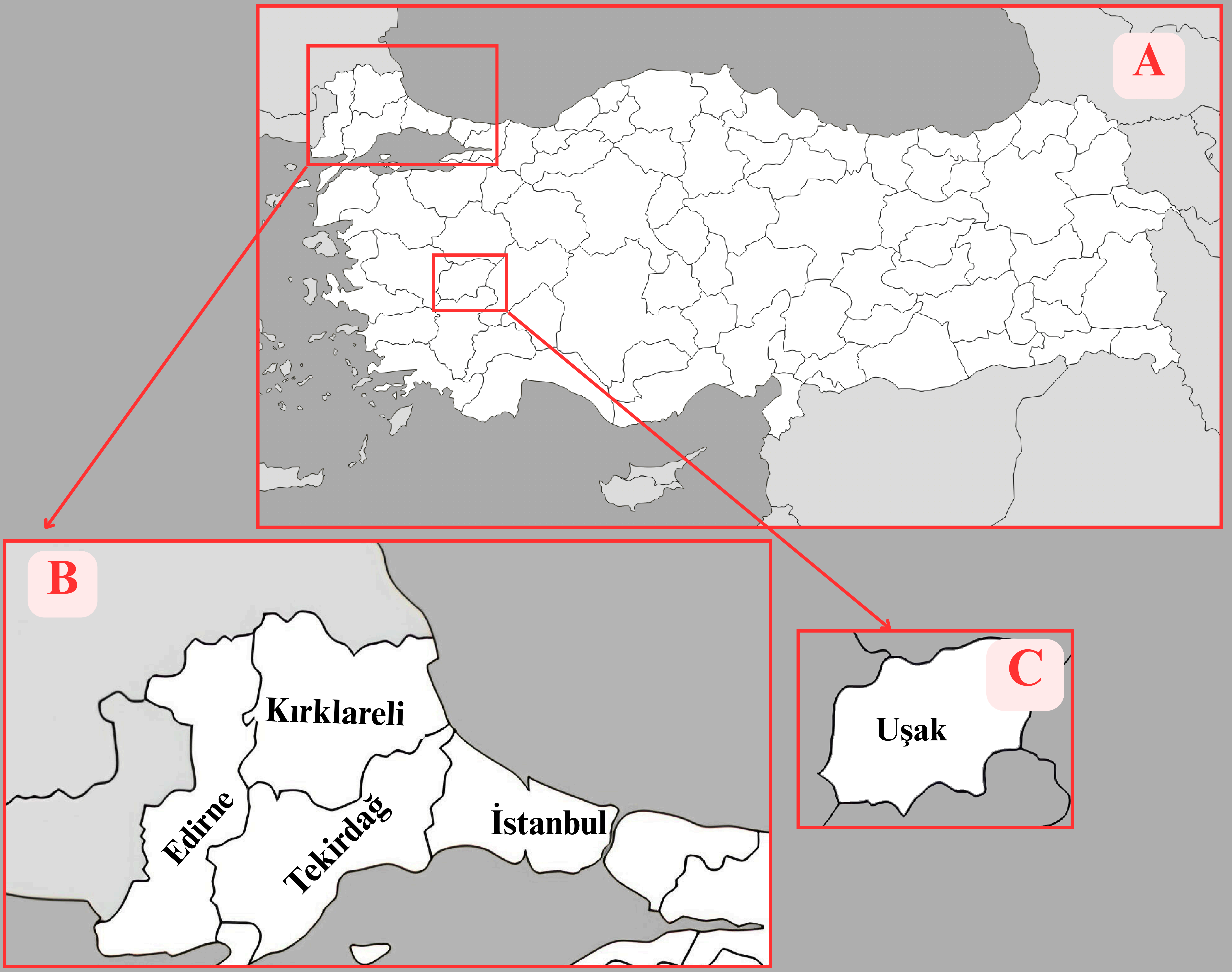

A: Map of Türkiye, B: Map showing the provinces sampled in the Thrace district (Edirne province n=45, Kırklareli province n=187, Tekirdağ province n=19, İstanbul province n=7), C: Uşak province n=23).
