# Supplementary material for: Molecular characterization of BCoV infecting vaccinated and non-vaccinated cattle in Thrace district Türkiye and isolation of field strains
Source: Virol J. 2025 Dec 1;22:388. doi: 10.1186/s12985-025-03010-3 (PMC12667072; doi:10.1186/s12985-025-03010-3)
Supplement: Supplementary file 2 — Additional file 2. Title of data: Supplementary Table S2. Description of data: BCoV sequences obtained from GenBank [file 12985_2025_3010_MOESM2_ESM.docx]

| Number of used BCoVs strain | Country | GenBank Accession Number | Strains |
| --- | --- | --- | --- |
| 1 | İtalia | EU814647.1 | Bovine coronavirusItaly-R |
| 2 | France | MG757143.1 | Bovine coronavirus France-U |
| 3 | Croatia | KM677177.1 | Bovine coronavirus Croatia-E |
| 4 | Sweden | KF169940.1 | Bovine coronavirus Sweden-E GIb |
| 5 | Iran | MK932862.1 | Bovine coronavirus Iran-E |
| 6 | Iran | MK932866.1 | Bovine coronavirus Iran-E |
| 7 | Croatia | KM677178.1 | Bovine coronavirus Croatia-E |
| 8 | Russia | OP652027.1 | Bovine coronavirus Russia-U |
| 9 | Denmark | KF169918.1 | Bovine coronavirus Denmark-R |
| 10 | Denmark | KF169919.1 | Bovine coronavirus Denmark-E |
| 11 | Türkiye | MK989623.1 | Bovine coronavirus Turkey-R |
| 12 | Poland | OL477656.1 | Bovine coronavirus Poland-R |
| 13 | France | D00731.1 | Bovine coronavirus France-E |
| 14 | Germany | EF193075.1 | Bovine coronavirus Germany-U GI |
| 15 | Korea | DQ389660.1 | Bovine coronavirus Korea-E-GIIa |
| 16 | USA | JQ741969.1 | Bovine coronavirus USA-E |
| 17 | China | KM985631.1 | Bovine coronavirus China-E |
| 18 | Korea | AY935645.1 | Bovine coronavirus Korea-E WD |
| 19 | USA | JQ741968.1 | Bovine coronavirus USA-R |
| 20 | USA | JX536392.1 | Bovine coronavirus USA-R |
| 21 | Modified Live Vaccine Strain | EU814648.1 | Bovine coronavirus Vaccine |
| 22 | Modified Live Vaccine Strain | JQ741970.1 | Bovine coronavirus CalfGard A609860-Vaccine USA-R |
| 23 | BCV Norden Vaccine Strain | M64668.1 | Bovine coronavirus Vaccine |
| 24 | BCV Killed Group-1b Vaccine Strain | OF134844.1 | KR 1020210038171-A/22-Korea-Vaccine |
| 25 | Reference Mebus Strain | U00735.2 | Bovine coronavirus Mebus strain GIa |
