# Supplementary material for: Molecular characterization of BCoV infecting vaccinated and non-vaccinated cattle in Thrace district Türkiye and isolation of field strains
Source: Virol J. 2025 Dec 1;22:388. doi: 10.1186/s12985-025-03010-3 (PMC12667072; doi:10.1186/s12985-025-03010-3)
Supplement: Supplementary file 3 — Additional file 3. Title of data: Supplementary Table S3: Description of data: Metadata compiled for all human and animal CoV reference sequences used in this study, including host species and country of origin, viral genus/subgenus, virus name, and GenBank accession numbers [file 12985_2025_3010_MOESM3_ESM.docx]

| Number of used CoVs strains | Host/Country | Genus/Subgenus | Virus | GenBank Accession Numbers |
| --- | --- | --- | --- | --- |
| 1 | Piglet/China | Betacoronavirus/ Embecovirus | PHEV_HEV-JT06 | EU919227.1 |
| 2 | Yak /China | Betacoronavirus/ Embecovirus | Yak_coronavirus_isolate | MH810157.1 |
| 3 | Mice/USA | Betacoronavirus/ Embecovirus | MHV-A59 | NP_045300.1 |
| 4 | Dromadary Camel/Morocco | Betacoronavirus/ Embecovirus | DcCoV_HKU23 | MN514971.1 |
| 5 | Horse/Japan | Betacoronavirus/ Embecovirus | ECoV | AB671299.1 |
| 6 | Dog/South Korea | Betacoronavirus/ Embecovirus | CRCoV | EU983107.1 |
| 7 | Rattus norvegicus )/China | Betacoronavirus/Embecobvirus | ChRCoV_HKU24 | YP_009113025.1 |
| 8 | Bat/China | Betacoronavirus/ Nobecovirus | BatCoV_HKU9 | YP_001039971.1 |
| 9 | Bat/China | Betacoronavirus/ Merbecovirus | BatCoV_HKU5 | YP_001039962.1 |
| 10 | Tylonycteris robustula (Bat)/ China | Betacoronavirus/ Merbecovirus | BatCoV_HKU4 | OM045061.1 |
| 11 | USA | Alphacoronavirus/Tegacovirus | FeCoV (FIPV) | YP_004070194.1 |
| 12 | Homo sapiens/Canada | Betacoronavirus/ Sarbecovirus | SARSCoV | YP_009825051.1 |
| 13 | Homo sapiens/China | Betacoronavirus/ Sarbecovirus | SARSCoV_2 | NC_045512.2 |
| 14 | Human (Child)/USA | Betacoronavirus/ Embecovirus | HECV_4408 | L07748.1 |
| 15 | Homo sapiens/Netherlands | Betacoronavirus/ Embecovirus | HCoV_OC43 | OK245433.1 |
| 16 | Homo sapiens /China | Betacoronavirus/ Embecovirus | HCoV_HKU1 | AAT98580.1 |
| 17 | Homo sapiens/UK | Betacoronavirus/ Merbecovirus | MERSCoV | AFY13307.1 |
| 18 | Cemal/Suudi Arabia | Alphacoronavirus/ Duvinacovirus/ HCoV-229E | Camel Alphacoronavirus | YP_009194639.1 |
