# Supplementary figures and images for: Molecular characterization of BCoV infecting vaccinated and non-vaccinated cattle in Thrace district Türkiye and isolation of field strains
Source: Virol J. 2025 Dec 1;22:388. doi: 10.1186/s12985-025-03010-3 (PMC12667072; doi:10.1186/s12985-025-03010-3)

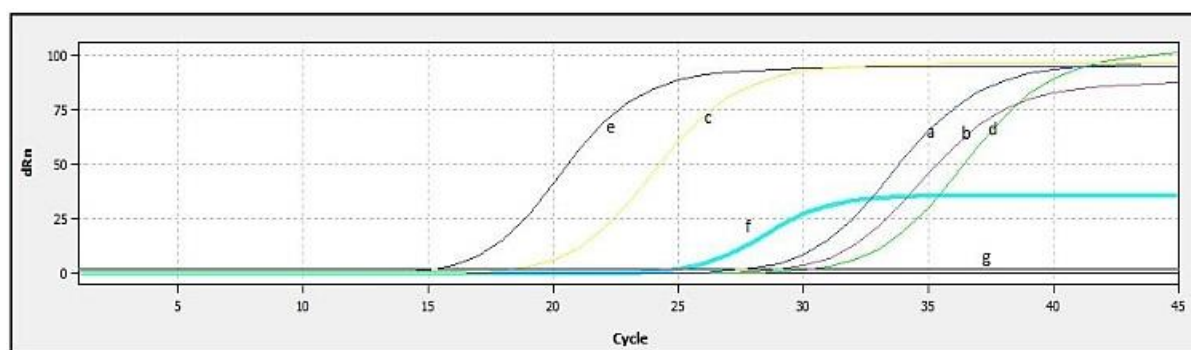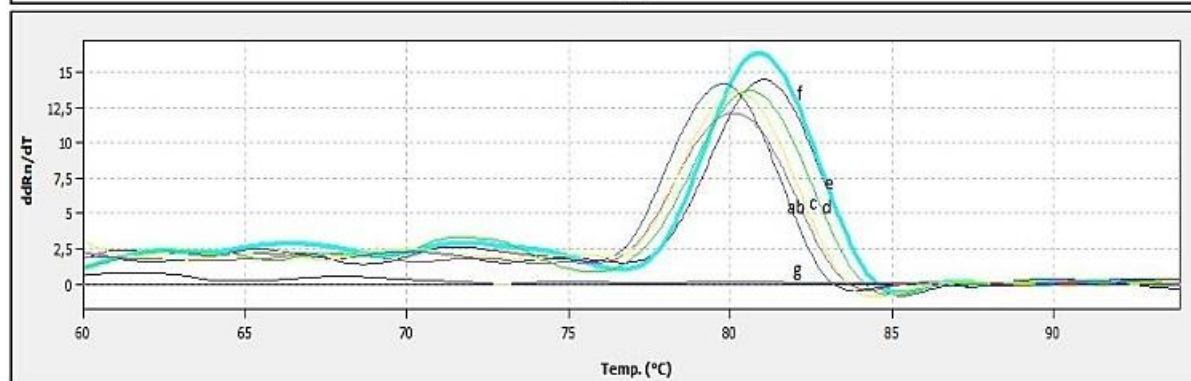

Supplement: Supplementary file 4 — Additional file 4. Title of data: Supplementary Figure S4. Description of data: Ct and melting curves of samples detected positive by SYBR-Green real-time RT-PCR. The letters given alphabetically indicate the sample codes. a:502/14-OS, b:1002/10-OS, c:1002/12-OS, d:2202/4-OS, e:1002/15-OS, f: BCoV positive control, g: negative control [file 12985_2025_3010_MOESM4_ESM.pdf]

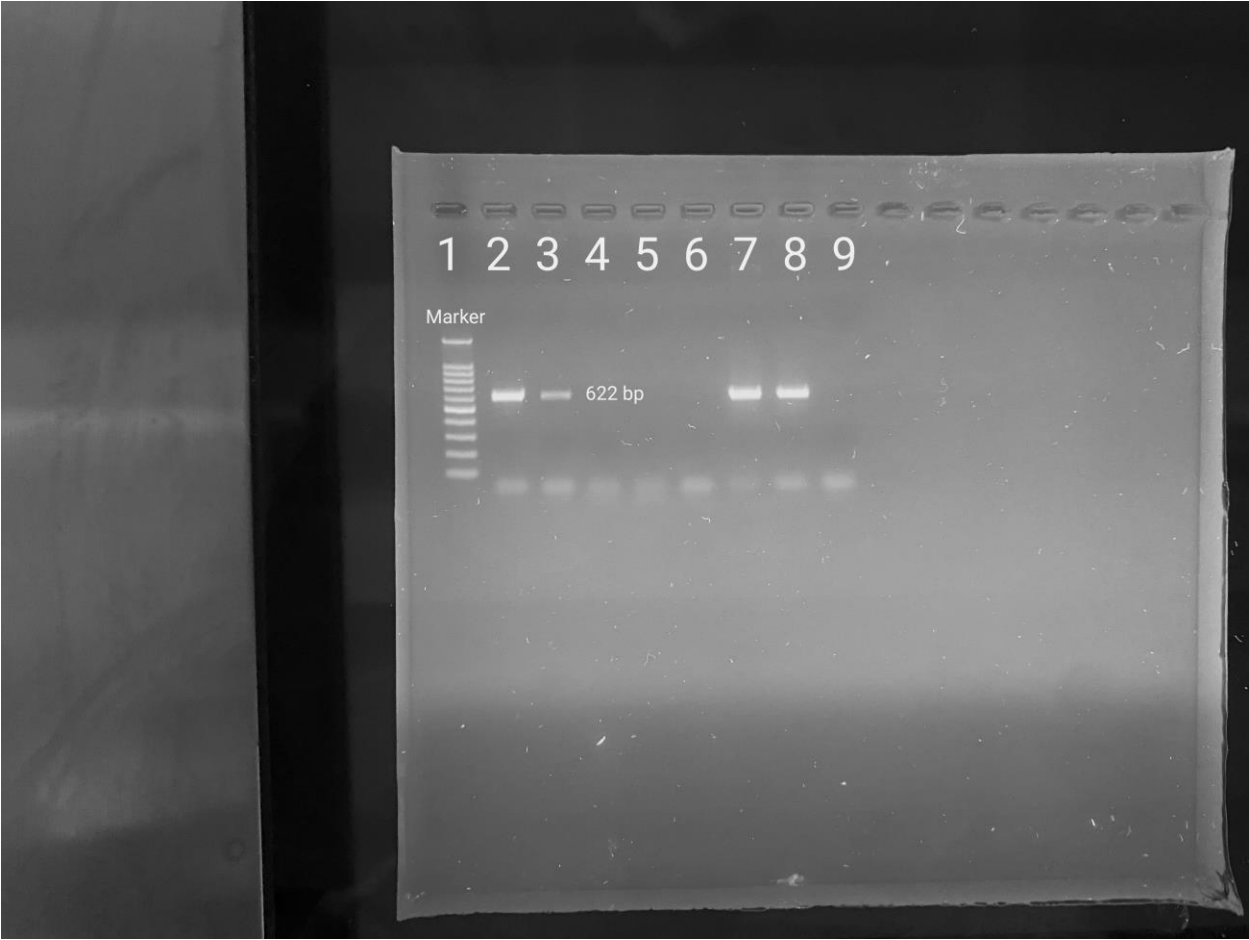

Supplement: Supplementary file 7 — Additional file 7. Title of data: Supplementary Figure S7. Description of data: Representative agarose gel showing the expected 622-bp BCoV S1 amplicon, including positive and negative field samples and positive and negative controls. 1: Marker. 2: 2601/19; 3: Positive control; 4: Negative control; 5: 2601/25; 6: 1401/4; 7: 2601/30; 8: 2402/11; 9: 502/14. Materials and reagents used for gel preparation and visualisation: Agarose, İnvitrogen, Cat. No: 16500100; TAE Buffer, Thermo Scientific, Cat. No:15558042; Loading Dye 6X, Thermo Scientific, Cat. No: R0611; Nucleic acid stain, Biomatik, Cat. No: A4205, Marker, Biomatik, Cat. No:M7123-100Loads) [file 12985_2025_3010_MOESM7_ESM.pdf]

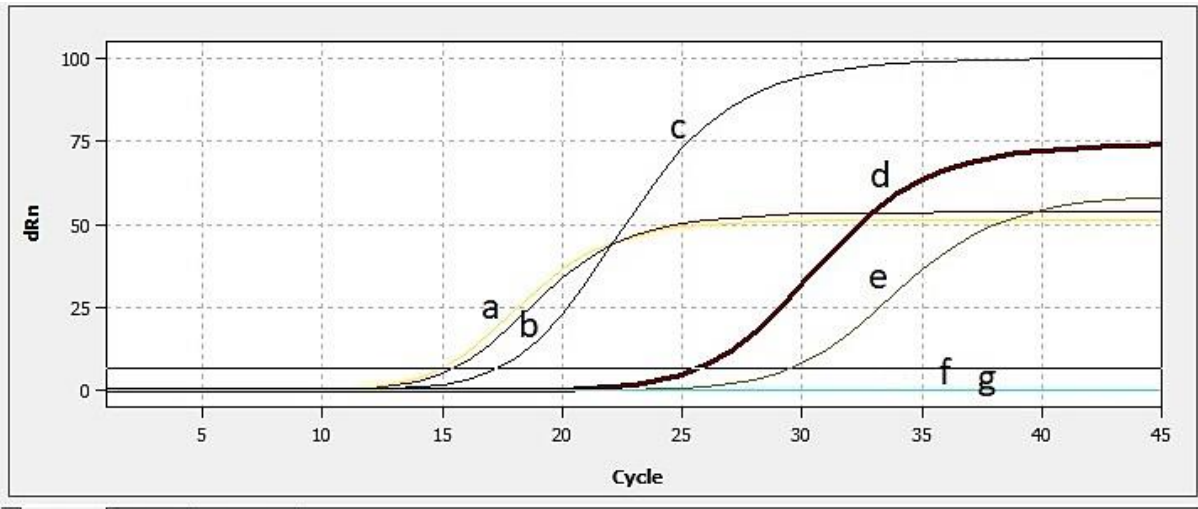

Supplement: Supplementary file 9 — Additional file 9. Title of data: Supplementary Figure S9. Description of data: real-time RT-PCR test results of samples taken from cell supernatants after the first inoculation and 1 st passage of the thesis samples. a: Ct of the 1 st passage of 1002-6/14.97, b: Ct of the 1 st passage of 2601-17/15.44, c: Ct of the 1 st inoculum of 1002-6/17.27, d: PCR positive control/Ct: 25.68, e: Ct of the 1 st inoculum of 2601-17/29.51, f: PCR negative control/Ct: no Ct, g: virus isolation control well/Ct: no Ct [file 12985_2025_3010_MOESM9_ESM.pdf]
