# Supplementary material for: Molecular characterization of BCoV infecting vaccinated and non-vaccinated cattle in Thrace district Türkiye and isolation of field strains
Source: Virol J. 2025 Dec 1;22:388. doi: 10.1186/s12985-025-03010-3 (PMC12667072; doi:10.1186/s12985-025-03010-3)
Supplement: Supplementary file 5 — Additional file 5. Title of data: Supplementary Table S5. Description of data: Chi-square analysis of BCoV positivity in samples from vaccinated and unvaccinated farms [file 12985_2025_3010_MOESM5_ESM.docx]

| PCR | Vaccinated | Unvaccinated | Total |
| --- | --- | --- | --- |
| Negative | 163 | 56 | 219 |
| Positive | 44 | 18 | 62 |
| Total | 207 | 74 | 281 |
|  |  |  |  |
| Chi-Square Value | | 0.147 | |
| DF | | 1 | |
| P Value | | 0.702 | |
