# Supplementary material for: Molecular characterization of BCoV infecting vaccinated and non-vaccinated cattle in Thrace district Türkiye and isolation of field strains
Source: Virol J. 2025 Dec 1;22:388. doi: 10.1186/s12985-025-03010-3 (PMC12667072; doi:10.1186/s12985-025-03010-3)
Supplement: Supplementary file 6 — Additional file 6. Title of data: Supplementary Table S6. Description of data: Descriptive data for farms with ≥1 PCR-positive sample, showing nasal/oropharyngeal and fecal swab results in vaccinated and non-vaccinated farms [file 12985_2025_3010_MOESM6_ESM.docx]

| **Vaccination Status of Farms Found Positive** | **Number of Nasal/Oropharyngeal Swabs Collected** | **Number of Positive Nasal/Oropharyngeal Swabs** | **Number of Fecal Swabs Collected** | **Number of Positive Fecal Swabs** |
| --- | --- | --- | --- | --- |
| **Unvaccinated Farms (34)** | 69 | 18 | 2 | 0 |
| **Vaccinated Farms (11)** | 114 | 23 | 71 | 21 |
